# Research on dynamic model construction method based on RecurDyn secondary development

**DOI:** 10.1371/journal.pone.0354073

**Published:** 2026-09-10

**Authors:** Lijuan Zhao, Shijie Yang, Yadong Wang, Xin Jin

**Affiliations:** 1 School of Mechanical Engineering, Liaoning Technical University, Fuxin, China; 2 The State Key Lab of Mining Machinery Engineering of Coal Industry, Liaoning Technical University, Fuxin, China; 3 Liaoning Province Large Scale Industrial and Mining Equipment Key Laboratory, Fuxin, China; University of Sharjah, UNITED ARAB EMIRATES

## Abstract

Efficient multibody dynamics (MBD) modeling is critical for the dynamic analysis of complex engineering equipment, yet conventional manual workflows remain labor-intensive, time-consuming, and error-prone. This paper proposes an automated MBD modeling methodology based on Python-driven meta-programming and automated C# code generation, with the cutting unit of a drum shearer selected as a case study. The proposed method first extracts Computer-Aided Design (CAD) assembly features and then employs a hybrid lexical-semantic dual-stream architecture with a multi-level gating mechanism for robust component identification. To improve fault tolerance under non-standard naming conditions, a pre-trained multilingual Transformer model is incorporated as an auxiliary semantic matching module. Subsequently, a customized execution engine invokes the RecurDyn ProcessNet (PNet) to automatically complete material assignment, topology integration, constraint and drive generation, and gear contact definition. To validate the methodology, kinematic simulations were conducted on three shearer cutting units with different structural complexities. Model reliability was further verified through rigid-flexible coupled Discrete Element Method–Multi Flexible Body Dynamics (DEM-MFBD) simulations and corresponding physical experiments on a shearer testing platform. Results show that the method requires less than 1% of traditional modeling time with high accuracy. The maximum relative error of transmission rotational speeds is only 0.025%. Furthermore, the time-domain Root Mean Square (RMS) error of the drum vibration acceleration between simulation and experiment is 4.8%, while the maximum relative error of the primary characteristic frequencies is 4.1%. This rule-driven methodology provides a transferable framework for the rapid modeling and simulation of diverse complex mechanical MBD systems.

## 1. Introduction

A mechanical multibody system is a complex system composed of multiple rigid or flexible bodies connected by kinematic pairs. Multibody system dynamics primarily studies the dynamic and kinematic characteristics of these interconnected bodies [1]. In engineering applications, virtual prototyping technology is commonly employed to simulate the dynamic behavior of such systems by constructing three-dimensional solid models and importing them into dynamic analysis software. This approach is widely applied in aerospace, automotive, machinery, power, and other engineering fields [2]. As the core executive component of a shearer operating under complex and harsh conditions, the cutting unit’s operational performance and reliability highly depend on the dynamic characteristics of its internal assembly. It is a typical mechanical multibody system. In response, numerous scholars at home and abroad have conducted extensive research on this topic. Lian et al. [3] developed a virtual prototype model of the shearer’s rocker arm using UG and ADAMS, and analyzed its dynamic characteristics by applying sinusoidal and step inputs to the shearer’s cutting components. Okolnishnikov et al. [4] developed an integrated simulation model of a longwall fully mechanized mining face and the shearer coal mining process using virtual prototyping technology and conducted simulation experiments to validate the model. Zhao et al. [5] developed a co-simulation platform integrating Pro/E, MATLAB, ANSYS, Adams, and Nsoft software. They established a rigid-flexible coupled model of the cutting components of a shearer for thin coal seams and performed dynamic simulations and fatigue life predictions. Hu et al. [6] conducted their study based on the MG1000/2500-WD shearer and obtained the working loads of its drum using LS-DYNA. Utilizing a simulation platform integrating HyperMesh, Mechanical APDL, and ADAMS, they developed a flexible multibody dynamics (MBD) model of the rocker arm to analyze its dynamic response and associated reliability issues. Li et al. [7] investigated the MG400/951-WD shearer by integrating rigid-flexible coupling technology with reliability sensitivity design theory and structural evolution algorithms to enhance its operational reliability. Tian et al. [8] created a 3D model of the shearer’s rocker arm using SolidWorks and established a rigid-discrete coupling simulation platform based on EDEM and ADAMS to analyze the deformation behavior of the shearer housing under complex loading conditions. With regard to the secondary development related to shearers, Zhang et al. [9] developed a parametric Computer-Aided Engineering (CAE) analysis system for shearer components by integrating Unigraphics NX secondary development tools with Visual Studio programming. Liu et al. [10] performed secondary development of SolidWorks, PFC3D, and Excel using C# on the Visual Studio 2022 platform. They developed a parametric system for cutting dynamics analysis and design of the shearer drum, enabling integrated parametric modeling and dynamic analysis. Zhang et al. [11] developed a high-precision three-dimensional simulation model of a complex coal seam containing gangue using EDEM secondary development technology. The model enabled dynamic correction and replacement of particle sets and was employed to obtain shearer cutting state information under various coal-rock conditions.

Despite significant advances in the construction, simulation, and optimization of dynamic models as outlined above, most current studies still depend on manual modeling and lack automation in the simulation modeling workflow. The manual modeling process is complex and error-prone, negatively impacting the efficiency and accuracy of simulation analyses. To address this issue, this study targets the shearer cutting unit as the engineering object and proposes an automated modeling method for mechanical multibody system dynamics based on Python-driven meta-programming and automated C# code generation. By extracting Computer-Aided Design (CAD) component features through semantic parsing, the method utilizes meta-programming techniques to automatically construct and compile C# programs based on the RecurDyn ProcessNet (PNet). To improve fault tolerance under non-standard naming conditions, a pre-trained multilingual Transformer model is incorporated as an auxiliary semantic matching module. This approach facilitates automated material assignment, topology integration, constraint and drive generation, and gear contact definition.

## 2. Foundations of automated modeling based on RecurDyn secondary development

### 2.1. Technical mechanism of RecurDyn PNet interface

RecurDyn provides robust secondary development capabilities, supporting both PNet-based customization and user subroutines tailored to the model-solving process [12]. PNet is a development tool used to automate workflows and enhance the functionality of RecurDyn. It enables users to create dynamic-link library (.DLL) within the Visual Studio integrated development environment (IDE), which can then be loaded and executed through the PNet Manager [13], thereby automating repetitive tasks such as modeling, simulation, and post-processing. Relevant C# code is written according to the design specifications, compiled into a.DLL, and subsequently called through the RecurDyn interface to complete the secondary development process.

This study employs PNet-based secondary development in RecurDyn to automate the modeling of the shearer cutting unit.

### 2.2. Methodology for the rapid modeling process

**Fig 1:**
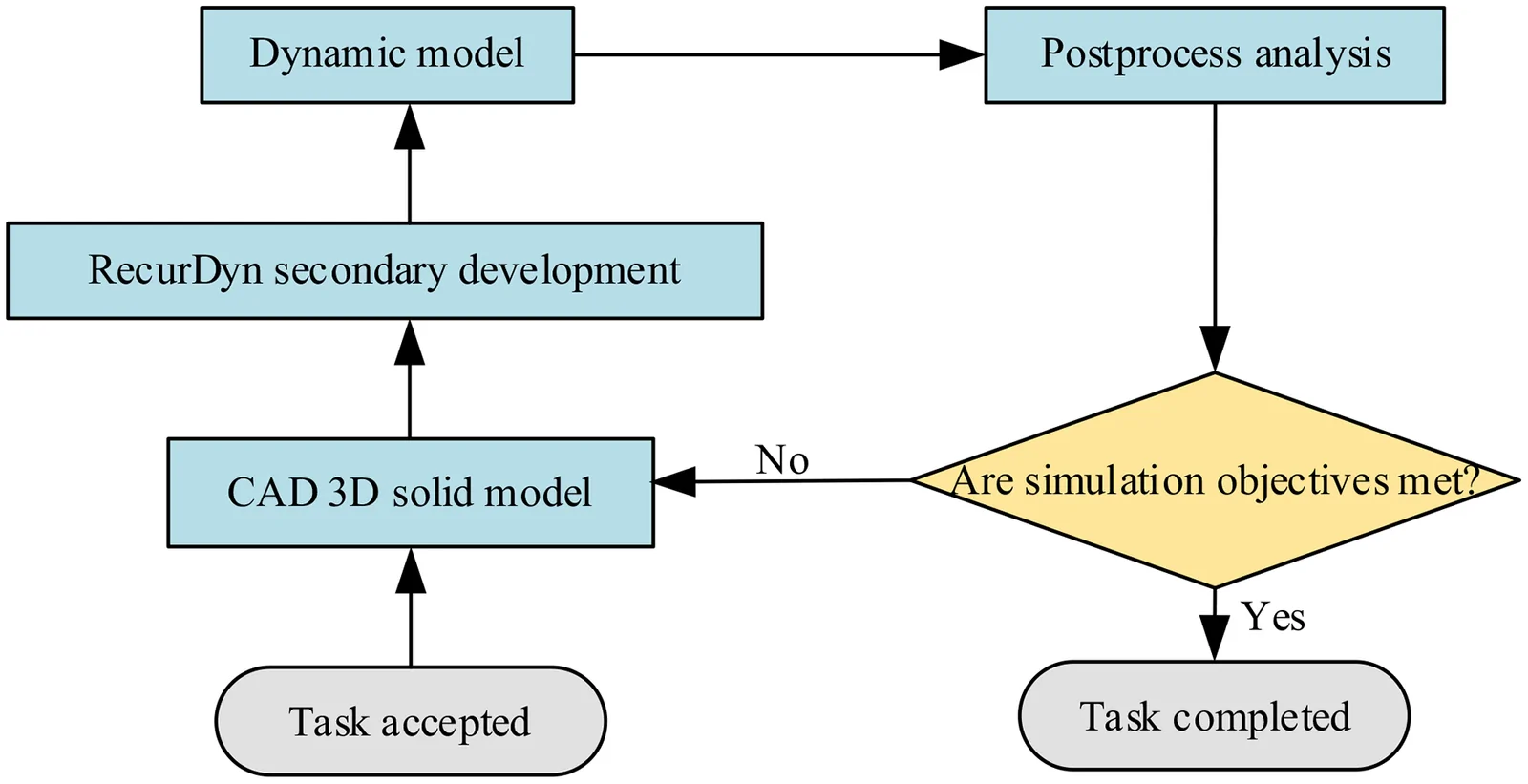
Automated modeling and simulation workflow based on secondary development.

The rapid dynamic modeling method and simulation workflow proposed in this study are illustrated in Fig 1. Rather than manually manipulating dynamic models within the RecurDyn interface, this approach achieves automation through the development and refinement of C# scripts. This code-driven operation significantly minimizes manual interactions and repetitive tasks, thereby enhancing both the efficiency and accuracy of the modeling process.

## 3. Overview of the drum shearer structure and establishment of the coal wall cutting model

### 3.1. Overview of the drum shearer structure

**Fig 2:**
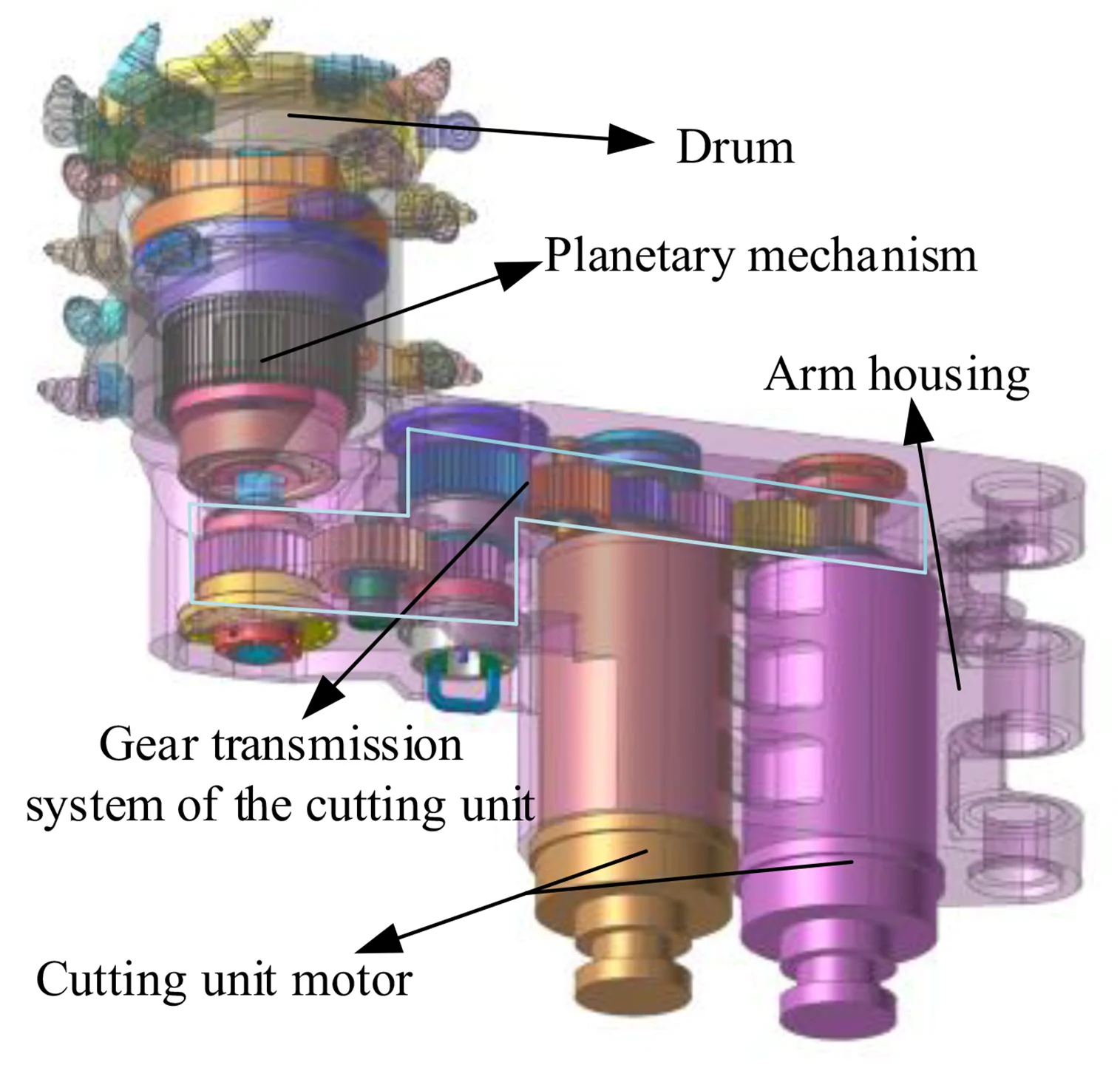
Inner structure of drum shearer.

Taking the MG2 × 55/250-BWD drum shearer cutting unit as an example, as shown in Fig 2, the cutting unit primarily consists of a drum, a planetary mechanism, a parallel-shaft cylindrical gear transmission system, and electric motors. The internal layouts of different cutting unit models vary. The arm housing, drum, and internal ring gear of the planetary mechanism are rendered transparently to facilitate the observation of the internal transmission structure. This model of cutting unit is driven by dual motors, with power transmitted through multi-stage cylindrical spur gears and the planetary mechanism. The drum is connected to the planetary carrier via a square-head coupling, thereby realizing the rotation-based coal and rock cutting function.

### 3.2. Construction of the discrete element model of the coal wall

**Fig 3:**
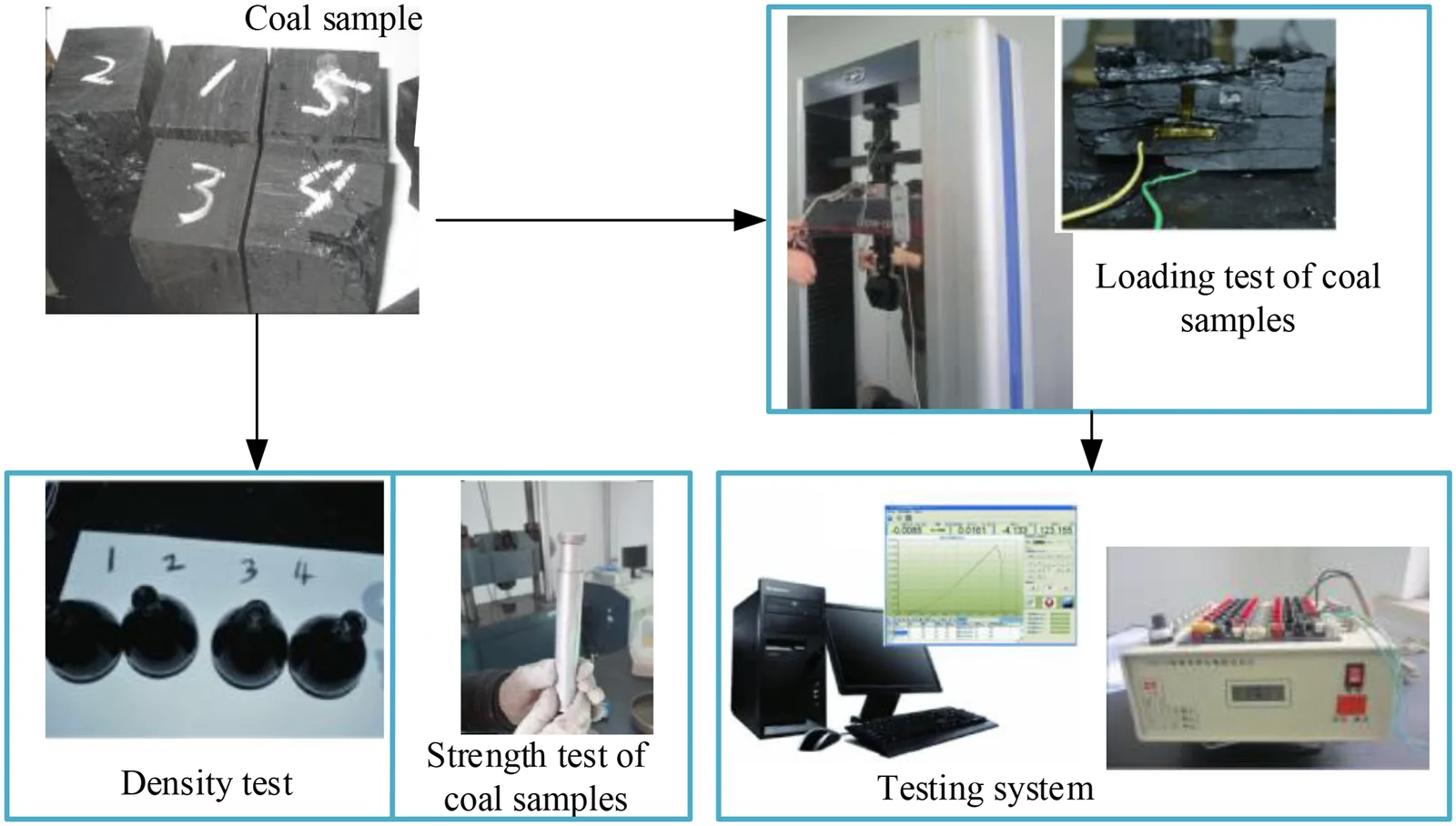
Test for determination of coal sample properties.

The accuracy of discrete element simulations highly depends on the precise calibration of material parameters. To establish a numerical model that can realistically reflect the mechanical response of the 17th coal seam in the Yanzhou mining area, laboratory tests were conducted on coal samples in accordance with standard sampling principles and testing specifications to obtain the necessary calibration parameters. The corresponding experimental procedures are illustrated in Fig 3, and the specific physical and mechanical properties of the coal samples obtained from the tests are listed in Table 1.

**Table 1:** Material parameters.

Materials	Elastic Modulus (MPa)	Density (kg/m^3^)	Poisson’s Ratio	Uniaxial Compressive Strength (MPa)	Consistent coefficient *f*
Coal	2010	1280	0.28	12	1.4

Considering the actual characteristics of coal particles, the bonding between particles was modeled using the Hertz-Mindlin with bonding contact model, while the contact between the particles and the shearer was defined as Hertz-Mindlin (no-slip) [14]. The bonding parameters between particles were determined through numerical uniaxial compression and Brazilian splitting tests [15], as summarized in Table 2.

**Table 2:** Bonding parameters between particles.

Name	Normal stiffness(N/m^3^)	Tangential stiffness(N/m^3^)	Normal maximum stress(Pa)	Tangential maximum stress(Pa)
Coal and Coal	1.2165 × 10^8^	9.7320 × 10^7^	8.3183 × 10^6^	2.3573 × 10^6^

**Fig 4:**
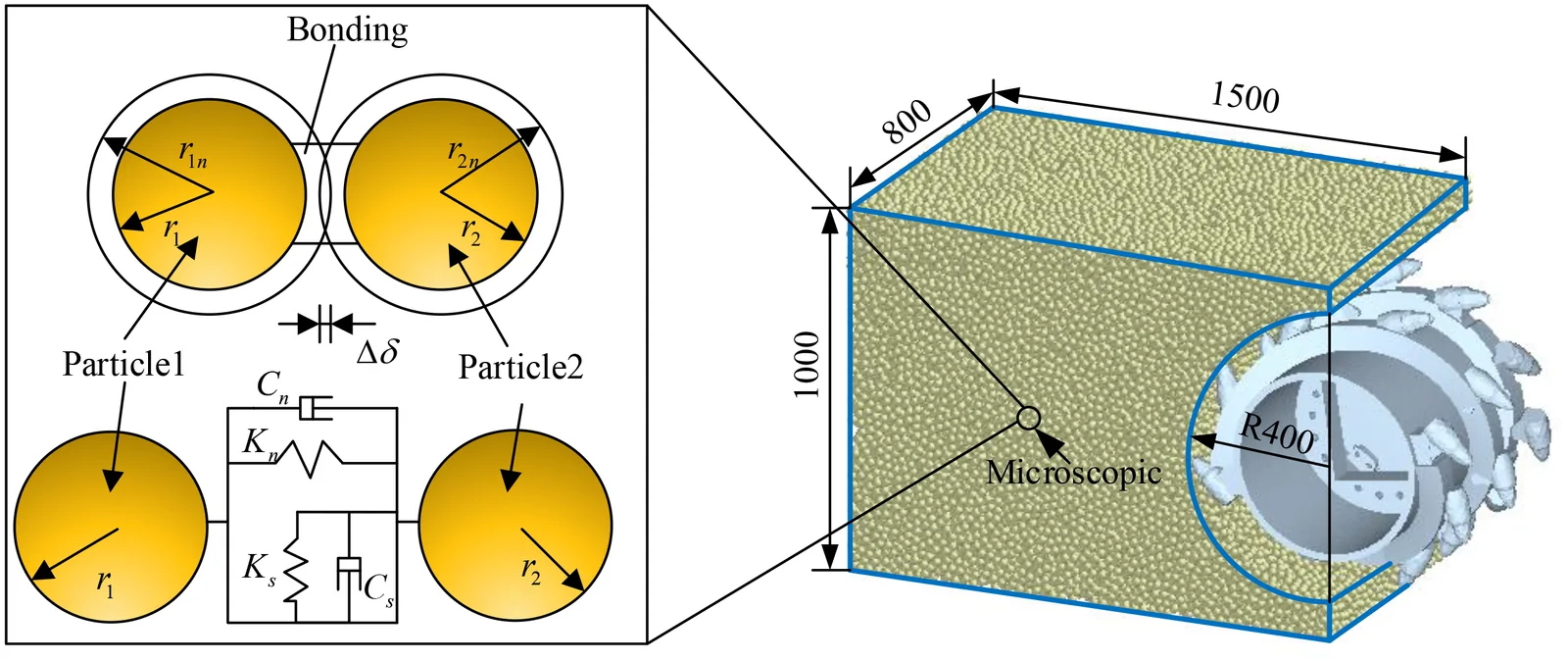
Virtual model of coal wall.

The coal seam thickness in Yanzhou Mine ranges from 500 mm to 1390 mm, with an average thickness of approximately 1000 mm. During model construction, the number of coal particles directly affects the simulation efficiency. To shorten the computation time while ensuring that the discrete element model can effectively perform the bidirectional coupling simulation of the shearer drum cutting the coal wall, the dimensions of the coal wall model were set to 1500 mm in length, 800 mm in width, and 1000 mm in height. A semicircular section corresponding to the drum radius was removed from the rectangular coal wall to construct its three-dimensional geometry, enabling the drum to rapidly enter a stable cutting state [16]. The coal wall was generated using particles with a radius of 12 mm, and the contact radius between particles was set to 14 mm. The coal wall model is illustrated in Fig 4.

## 4. Automated process for dynamic modeling of the shearer cutting unit

**Fig 5:**
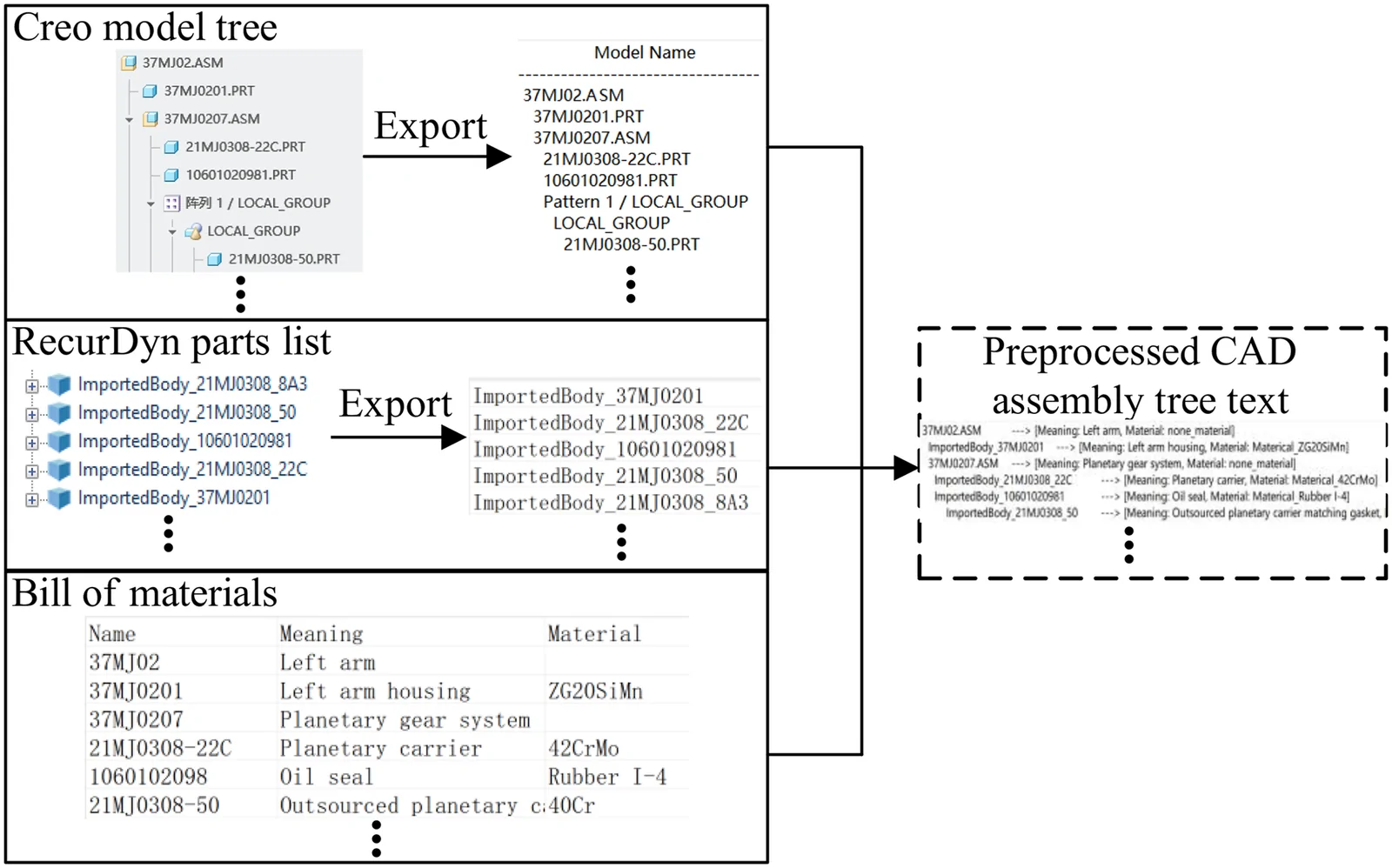
Data fusion and preprocessing workflow for CAD assembly structures.

The rapid construction of a dynamic model of the shearer cutting unit requires the prior creation of a geometric model using CAD modeling software. This model can be imported into RecurDyn through neutral 3D file formats such as X_T, IGES, or STEP. In this study, the model was built using a STEP-format neutral file. To address the semantic gaps and topology reconstruction challenges between complex 3D CAD assembly models and MBD simulation software, this paper proposes an automated modeling methodology based on hierarchical topology parsing, heuristic rules, and dynamic code generation. Taking pre-processed CAD assembly tree text as input, the methodology achieves automated construction with minimal manual intervention of MBD models for complex shearer cutting units through hybrid semantic alignment, hierarchical graph topology derivation, and dynamic code generation technologies. The preprocessed CAD assembly tree text is synthesized via a Python-based pipeline that integrates three primary data sources: enterprise Bills of Materials (BOM) (comprising part numbers, descriptions, and material specifications), assembly tree structures from CAD software (e.g., Creo), and part lists generated within RecurDyn after importing neutral STEP files. The synthesis result is illustrated in Fig 5.

### 4.1. Graph representation and parsing of assembly hierarchies

Traditional CAD models are inherently structured as top-down hierarchical trees. To facilitate the computational interpretation and processing of assembly relationships, this paper first maps the preprocessed CAD assembly tree text onto a rigorous Directed Acyclic Graph (DAG), the formal representation of which is defined in Eq. (1).


$$\begin{array}{c} G=\left(V,E\right) \end{array}$$ (1)

Where the vertex set $V=\{v_{\text{1}},v_{\text{2}},...,v_{n}\}$ represents the various sub-assemblies or individual parts within the assembly, and the edge set *E* denotes the parent-child dependencies between the nodes.

For each input line in the preprocessed text, the algorithm extracts the topological depth *D*(*v*_*i*_) based on the indentation level. Each node *v*_*i*_ primarily consists of the entity name *N*(*v*_*i*_), original semantics *M*(*v*_*i*_), and material name *P*(*v*_*i*_). The parsing algorithm dynamically maintains an Active Node Stack; upon detecting a new node *v*_*j*_, it automatically establishes directed edges by comparing *D*(*v*_*j*_) with the depth of the top node in the stack, thereby constructing a comprehensive global assembly data structure. This structure preserves the original physical assembly hierarchy of the machine system. Building upon this, the system leverages Python-driven meta-programming and automated C# code generation to perform secondary development of RecurDyn via the PNet platform. This workflow establishes the logical foundation for the automated dynamic modeling of the shearer cutting unit.

### 4.2. Fault-tolerant component identification based on hybrid lexical-semantic matching

Within the automated modeling methodology for complex mechanical equipment, the proposed system predominantly leverages semantic descriptors from the BOM to map and pinpoint the leaf-level 3D entities within the CAD assembly tree. However, real-world industrial BOM texts exhibit a high degree of non-standardization and inconsistency. This phenomenon is primarily driven by manual entry errors (such as typographical omissions and misspellings), non-standard industry abbreviations, and hybrid Chinese-English content. Relying solely on exact string matching is highly susceptible to minor textual imperfections, which can easily lead to the identification failure of core components. This, in turn, results in the disruption of the kinematic topological chain. Furthermore, original CAD assemblies typically contain a large number of secondary components (e.g., micro-fasteners, and seals) that have a negligible impact on macroscopic dynamics. Incorporating the complete set of nodes into the simulation engine would lead to a substantial increase in the degrees of freedom (DOF) of the MBD model, thereby significantly reducing simulation efficiency.

**Fig 6:**
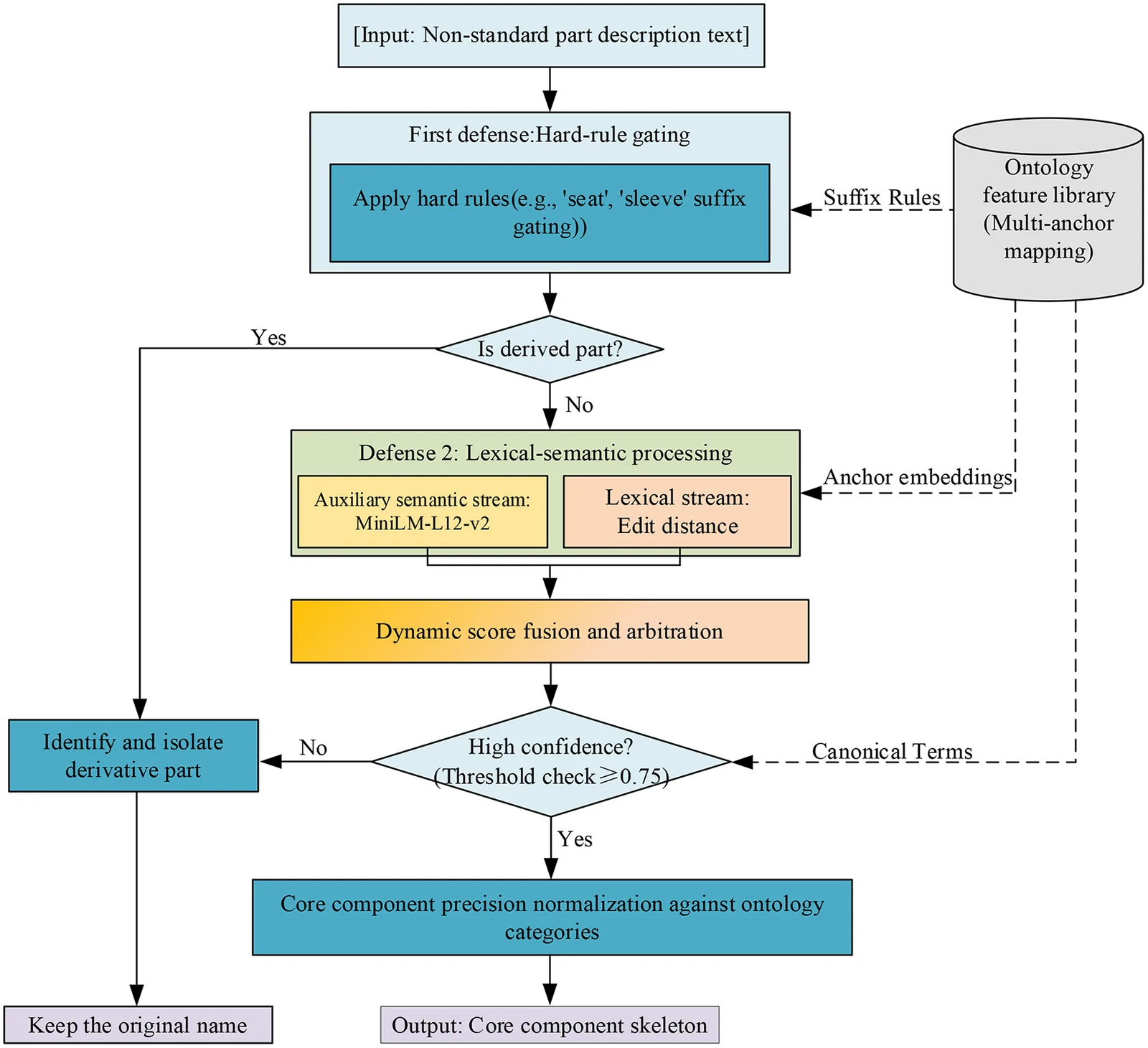
Architecture of the fault-tolerant component identification strategy based on hybrid lexical-semantic matching and multi-level gating.

To address these challenges, this paper proposes a fault-tolerant component identification strategy based on hybrid lexical-semantic matching and multi-level gating. Initially, the system constructs an ontology feature library grounded in multi-anchor mapping and introduces a negative constraint gating mechanism based on blacklisted affixes as the first line of defense. This defensive layer employs hard-rule-based prioritized screening to precisely identify components with suffixes such as “-sleeve,” “-housing,” or “-cover” as derivative accessories. For instance, it isolates the “bearing housing” from the core transmission component “bearing,” thereby eliminating semantic ambiguity in the mechanical domain at its source. For texts that bypass the initial rule-based filtering, the system invokes the second line of defense. Specifically, a pre-trained multilingual sentence-embedding model based on a lightweight Transformer architecture (paraphrase-multilingual-MiniLM-L12-v2) is incorporated as an auxiliary semantic matching module. It evaluates the cosine similarity between non-standard component descriptions and predefined ontology terms. This mechanism improves the identification tolerance for cross-linguistic abbreviations and ambiguous expressions. Simultaneously, a lexical verification stream based on edit distance is introduced to identify minor spelling errors that may not be reliably captured by semantic similarity matching. Finally, the system employs a dynamic high-score arbitration mechanism with a confidence threshold set at 0.75 to accurately normalize non-standard short texts into predefined core component categories. The methodology effectively preserves the core skeleton while physically segregating and ignoring secondary derivative parts, thereby substantially reducing the complexity of the kinematic topological chain. The underlying mechanism is illustrated in Fig 6. To validate the fault tolerance of the identification strategy under non-standard naming conditions, various types of textual interference noise were artificially introduced for testing. The specific recognition performance is presented in Table 3.

**Table 3:** Recognition results for noisy inputs.

BOM Input (Noisy)	Identified Component	Decision Mechanism	Score
shatf	Shaft	Lexical Edit Distance	0.8000
plnet carir	Planet Carrier	Lexical Edit Distance	0.8800
bering	Bearing	Lexical Edit Distance	0.9231
Drumm	Drum	Semantic Similarity Matching	0.9325
transmission axle	Drive Shaft	Semantic Similarity Matching	0.8501
drive motor Y160M-4	Motor	Exact Rule Match	1.0000
II-sun gearr	Stage-2 Sun Gear	Exact Rule Match	1.0000
Level-3 sun gear	Stage-3 Sun Gear	Exact Rule Match	1.0000

### 4.3. Automated material assignment mechanism based on attribute mapping

**Fig 7:**
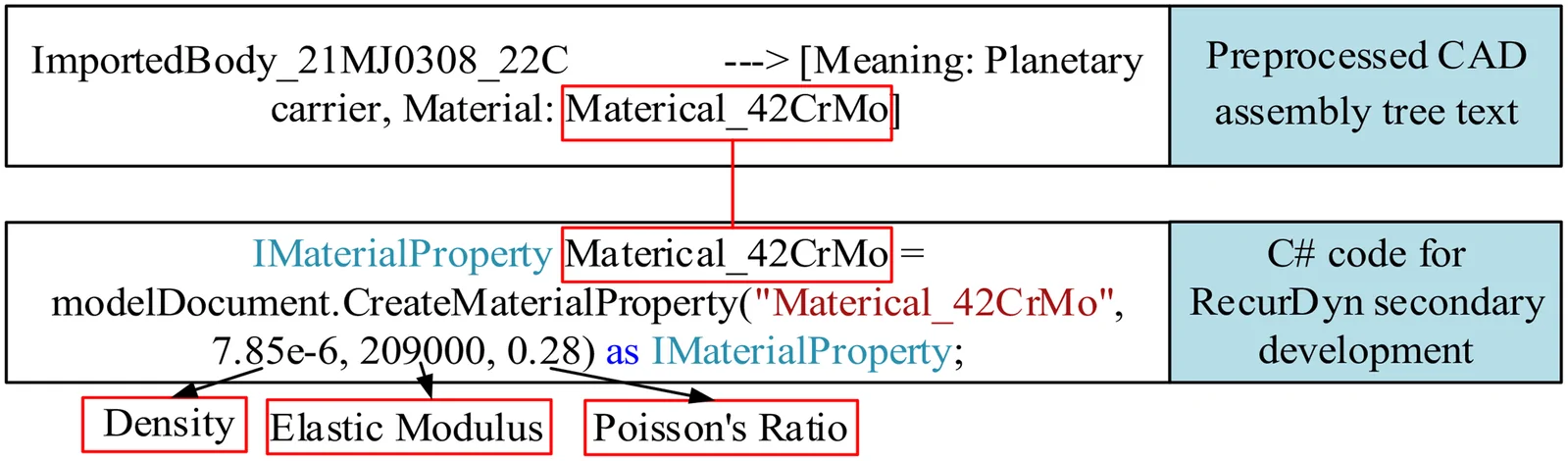
Automated material mapping and code generation.

To ensure the physical consistency of the dynamic simulation, the system establishes a material database based on RecurDyn, comprising density, Poisson’s ratio, and Young’s modulus. To address the lack of physical attributes in imported CAD models, the proposed algorithm implements automated material mapping by parsing assembly tree text and utilizing keyword-based heuristic matching. Taking the planetary carrier as an example, the principles of its mapping and code generation are illustrated in Fig 7.

### 4.4. Topological fusion of complex components

**Fig 8:**
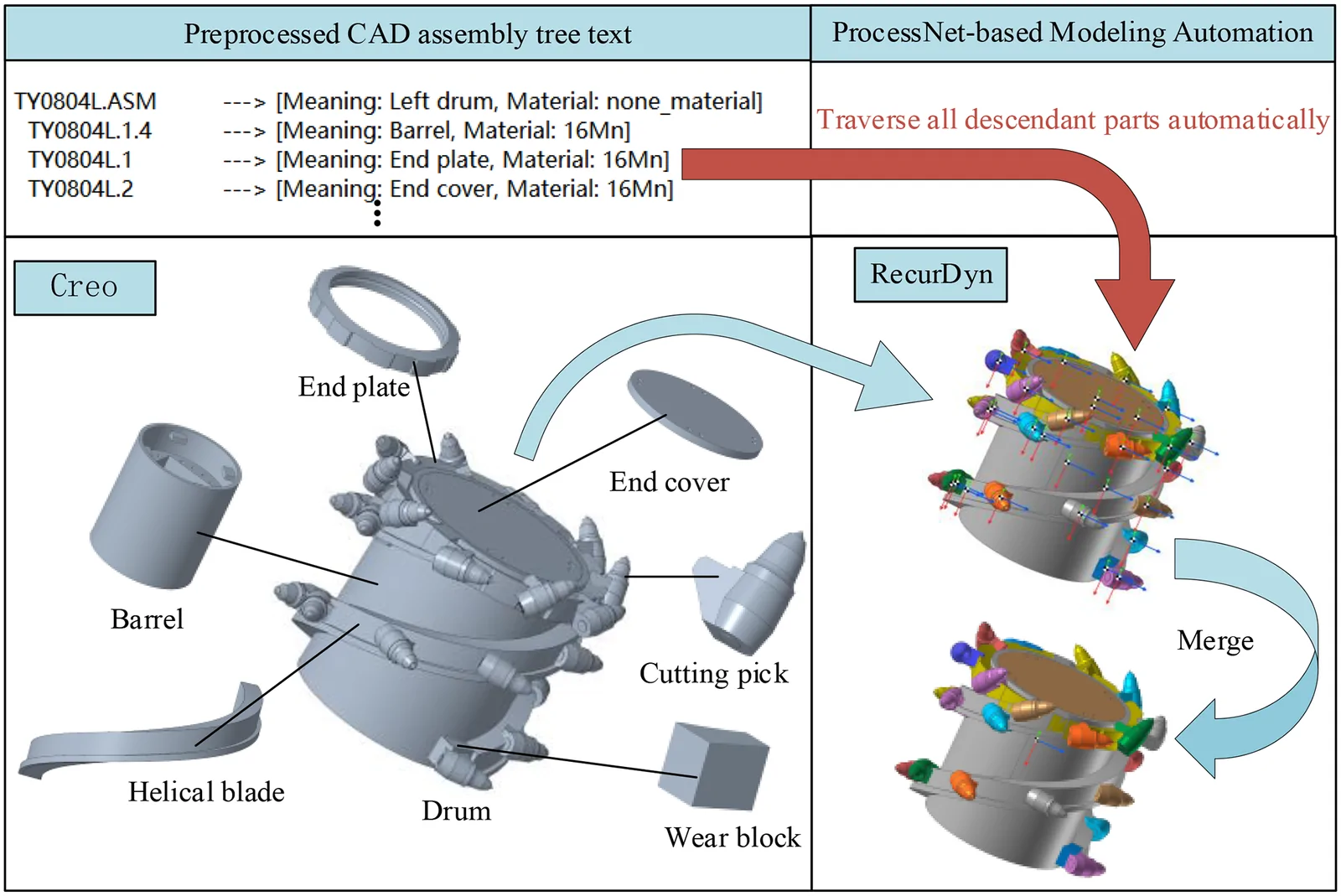
Automated fusion processing workflow for the drum assembly.

The shearer cutting unit features an intricate structure, particularly the drum assembly, which comprises numerous picks, spiral vanes, and end disks. Retaining the original topological state in MBD simulations would lead to prohibitive computational overhead if the full-scale model were imported. To this end, adjacent components with no internal relative motion and those not requiring intensive study are unified and merged into a single independent rigid body [17]. During the fusion of the drum assembly, the program initially retrieves the preprocessed assembly tree structure and precisely pinpoints the.ASM root node characterized by “drum” semantics. Subsequently, a Depth-First Search (DFS) algorithm is employed to recursively traverse and automatically merge all subordinate hierarchical parts. This facilitates the automated integration of the drum assembly, the processing principle of which is illustrated in Fig 8.

**Fig 9:**
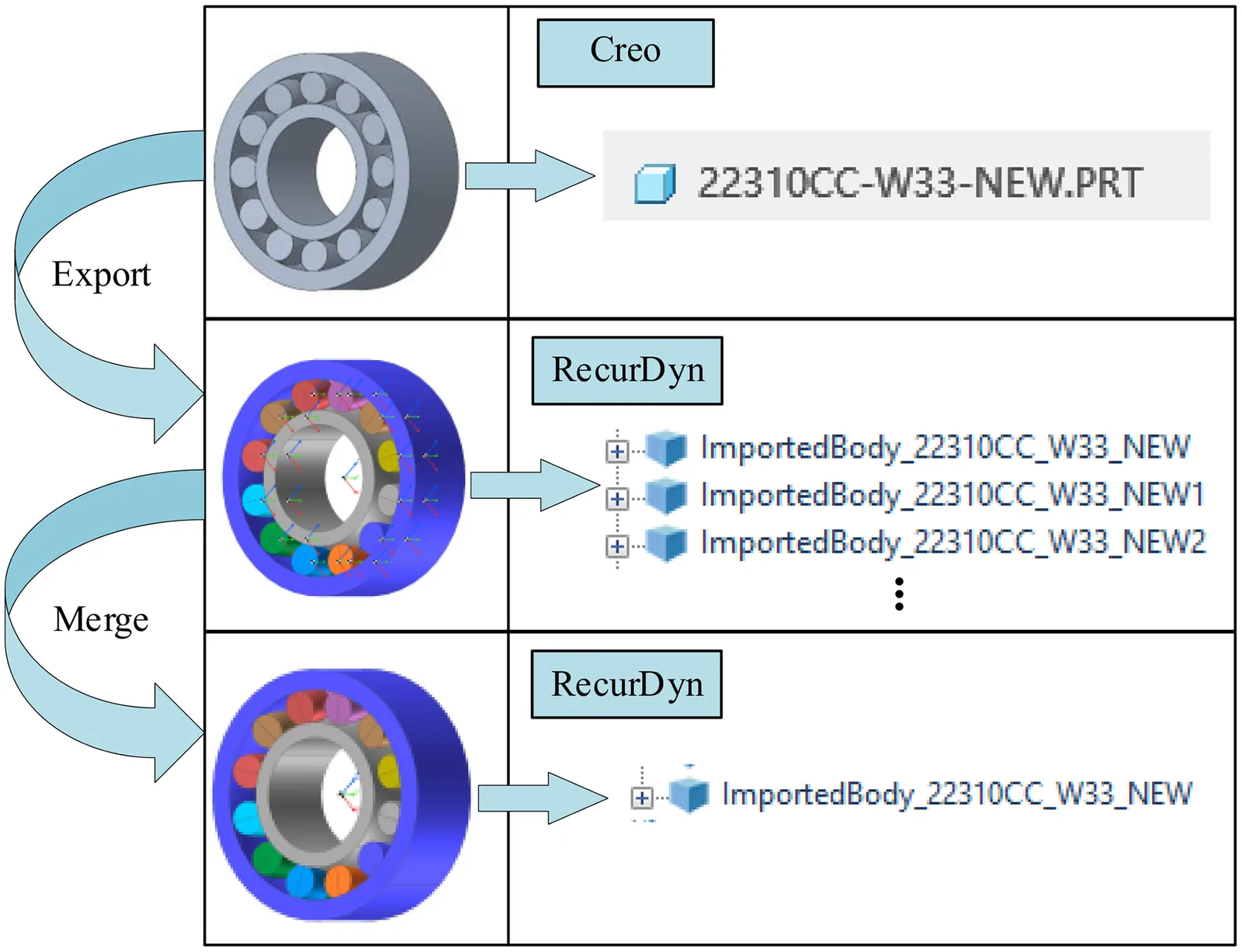
Automated fusion processing workflow for the bearing.

In simulation studies where internal bearing dynamics are not the primary focus, bearings within the shearer cutting unit are typically treated as integrated rigid bodies [18–20]. However, due to the discrepancy in geometry recognition mechanisms between Creo and RecurDyn, a bearing encapsulated as a single part (.PRT) in Creo is frequently discretized into multiple independent entities upon importation into RecurDyn. The root cause of this phenomenon is that RecurDyn’s default identification logic treats geometries without volumetric interference as distinct physical objects. Since the interaction between the rollers and the raceways (inner and outer rings) typically involves point or line contact rather than volumetric overlap, they are perceived by the system as discrete individuals. To resolve this, the proposed code strips prefixes and numerical suffixes from the imported part names to precisely extract the “feature signature” of the bearing, subsequently merging the associated components into a single entity within RecurDyn. The synthesis principle of these bearing entities is illustrated in Fig 9.

### 4.5. Constraint and drive configuration of the cutting unit

**Fig 10:**
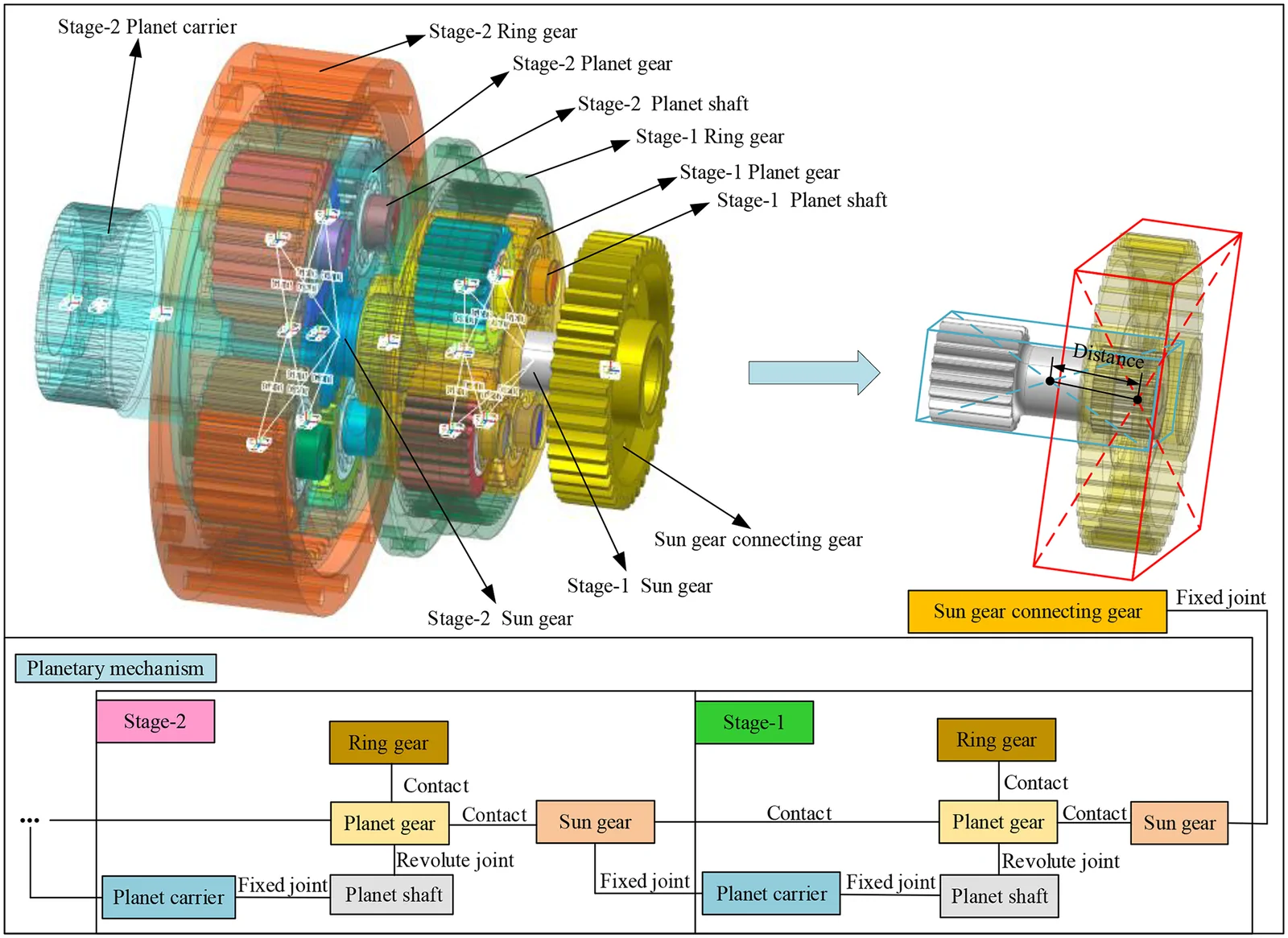
Constraint configuration and topological modeling of multi-stage planetary transmission systems.

The transmission chain of the shearer cutting unit is typically composed of multi-stage parallel shafts and planetary mechanisms. To address the variable stages of planetary gear trains and the complexity of their components, this paper proposes an adaptive modeling algorithm. By parsing assembly hierarchies and topological names, the algorithm automatically determines the number of planetary stages and identifies core components, such as sun gears, planet gears, and planetary carriers. For the constraint mapping of sun gears and adjacent gears across different sub-assemblies, where traditional topological path searching often fails to achieve effective matching, this study introduces a criterion based on the minimization of spatial geometric center deviation. By calculating the distances between geometric centers, the algorithm precisely identifies the optimal connection pairs and automatically generates Fixed Joints. The principle is illustrated in Fig 10.

**Fig 11:**
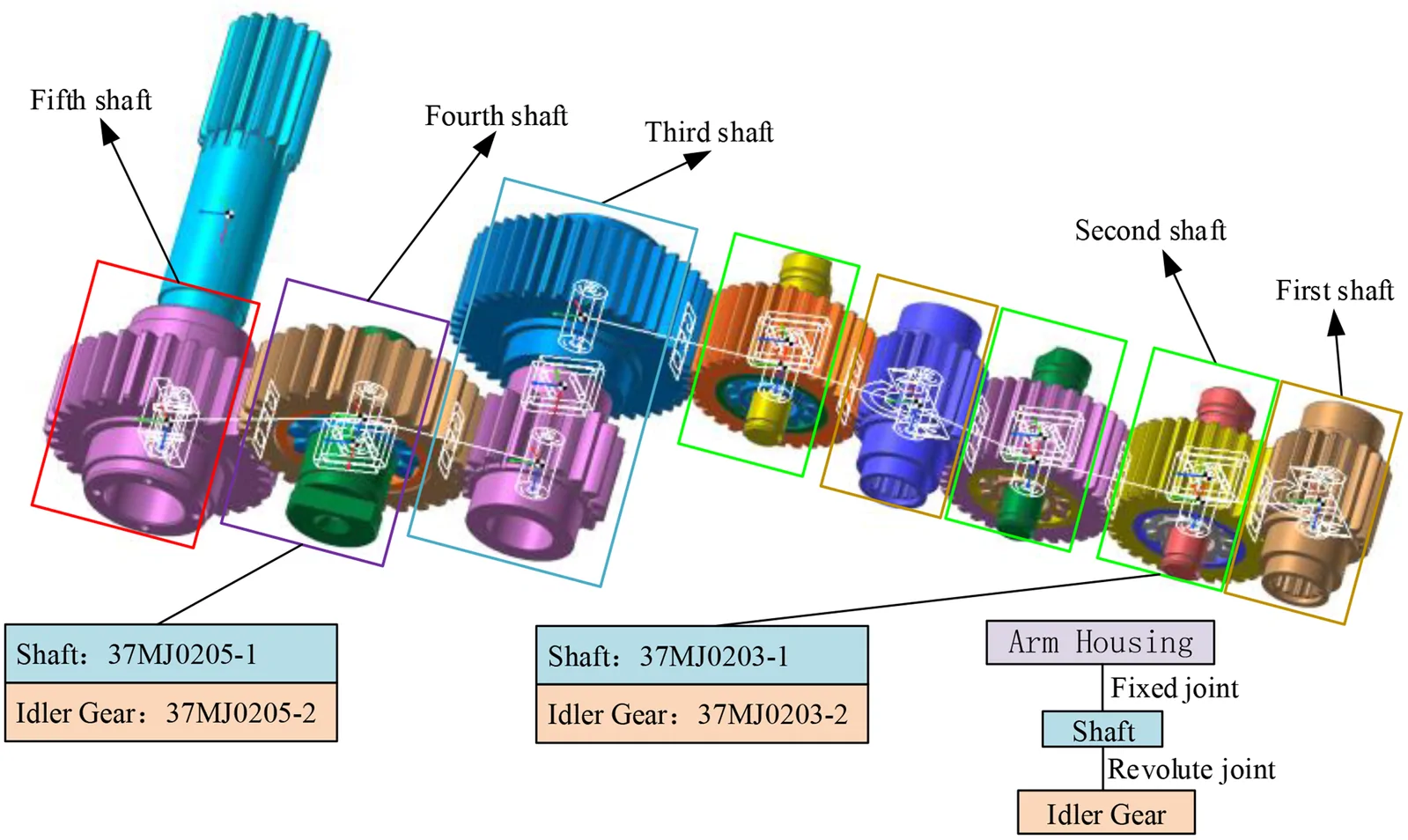
Schematic diagram of transmission component pairing and constraint topology.

The cutting unit of a shearer typically employs a multi-stage parallel-axis cylindrical gear transmission. Taking the MG2 × 55/250-BWD model as an example, its driveline consists of five distinct shafts, as illustrated in Fig 11. To address the typical “multi-instance semantic ambiguity” problem, where multiple entities share identical semantic labels such as “idler gear” or “shaft”, traditional classification methods often fail to establish precise assembly mappings, leading to topological mismatches. To mitigate this, this paper proposes an adaptive pairing mechanism based on naming rule similarity. Building upon semantic dimensionality reduction and set partitioning, the mechanism achieves precise binding between a specific idler gear (e.g., 37MJ0205−2) and its exclusive supporting shaft (e.g., 37MJ0205-1) by calculating the string similarity of their part numbers. Once the ambiguity is resolved, the program strictly follows a preset assembly topological chain to apply constraints: a Fixed Joint is first generated between the ranging arm housing and the shaft, followed by a Revolute Joint between the idler gear and the shaft. This mechanism has been extended to all associated nodes with pairing ambiguities, ensuring high precision and automation in constraint construction.

The rotational motion is applied to the revolute joint of the first-axis shaft gear within the cutting unit. To simulate the motor drive function, a Step function is employed, which utilizes a cubic polynomial to approximate the Heaviside step function. Its mathematical definition is expressed as Eq. (2) following [21]:


$$\begin{array}{c} \text{Step}\left(x,x_{\text{0}},h_{\text{0}},x_{\text{1}},h_{\text{1}}\right)=\left\{ \begin{array}{cc} h_{\text{0}}, & x\leq x_{\text{0}}\\ h_{\text{0}}+(h_{\text{1}}-h_{\text{0}}){\left[\frac{\left(x-x_{\text{0}}\right)}{\left(x_{\text{1}}-x_{\text{0}}\right)}\right]}^{\text{2}}\left\{3-2\frac{\left(x-x_{\text{0}}\right)}{\left(x_{\text{1}}-x_{\text{0}}\right)}\right\}, & x_{\text{0}}<x<x_{\text{1}}\\ h_{\text{1}}, & x\geq x_{\text{1}} \end{array}\right. \end{array}$$ (2)

Where *x* is the independent variable, defined as time in this study; *x*_*0*_ and *x*_*1*_ are the initial and final values of the independent variable for the step function, respectively; and *h*_*0*_ and *h*_*1*_ represent the corresponding function values at the start and end points.

### 4.6. Automated configuration of gear contact relationships based on semantic and spatial heuristics

The automatic establishment of contact relationships is categorized into two modes: targeted matching and spatial searching. For planetary mechanisms with distinct structural features, the algorithm directly establishes specific geometric contact pairs among the sun gear, planet gears, and internal ring gear via semantic identification; the corresponding settings are illustrated in the “contact” portion of Fig 10. Conversely, for parallel-axis gear sets with disordered non-planetary layouts in the shearer cutting unit, this paper proposes a contact reconstruction algorithm based on bounding box spatial features and 1D clustering.

Firstly, the 3D bounding boxes of all parallel-axis gears are extracted, and their geometric center coordinates, *P*_*i*_ = (*x*_*i*_, *y*_*i*_, *z*_*i*_), are calculated. Leveraging the topological characteristic that parallel-axis transmission systems are arranged in a specific axial sequence, the algorithm sorts the gears based on their z-axis coordinates. Gears with a coordinate difference within a tolerance threshold of 1 mm are clustered into the same shaft set to enhance robustness against initial assembly errors in CAD models. Following shaft classification, the algorithm infers the actual meshing pairs between adjacent shaft sets by minimizing Euclidean distances. Specifically, for the *k*-th shaft set *G*_*k*_ and the (*k* + 1)-th shaft set *G*_*k+1*_, a global search is performed using Eq. (3) to identify the gear pair with the minimum distance *d*(*u*,*v*).


$$\begin{array}{c} d\left(u,v\right)=\sqrt{(x_{u}-x_{v}{)}^{\text{2}}+(y_{u}-y_{v}{)}^{\text{2}}+(z_{u}-z_{v}{)}^{\text{2}}} \end{array}$$ (3)

Where *u* and *v* are the candidate gear entities within the adjacent shaft sets *G*_*k*_ and *G*_*k+1*_, respectively.

**Fig 12:**
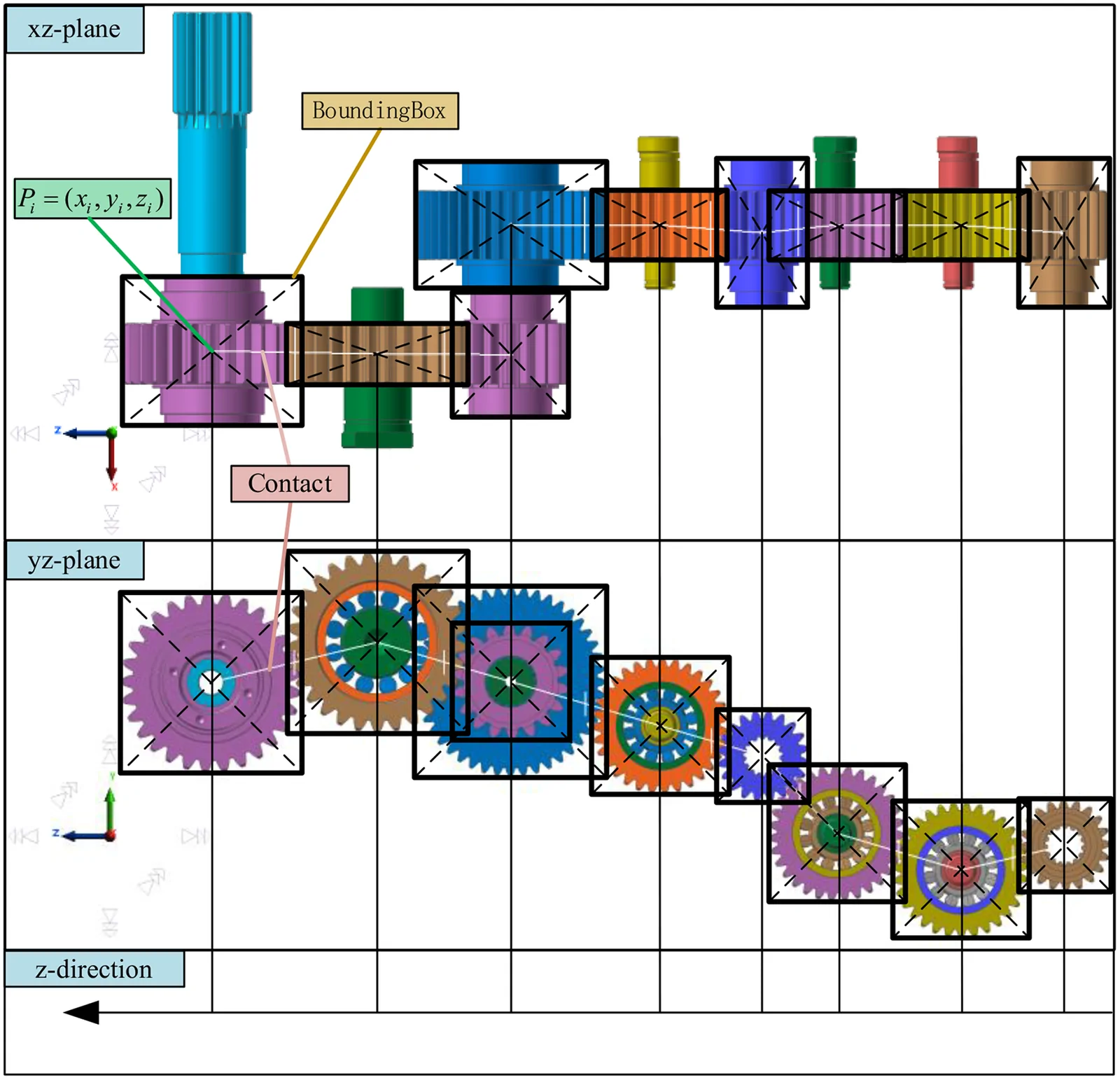
Schematic diagram of automated modeling for gear transmission chains.

After pinpointing the optimal proximity pairs, the system automatically invokes the Application Programming Interface (API) to generate high-precision contact constraints on the mesh surfaces of the corresponding entities, as shown in Fig 12. This strategy requires no manual intervention and can robustly transform discrete geometric models into dynamic topological structures with closed-loop transmission logic, effectively enhancing both the efficiency and precision of modeling complex gear transmission chains.

### 4.7. Redundant feature removal

To further optimize computational costs, small-scale components such as bolts, washers, and non-load-bearing fasteners contribute negligibly to the macroscopic dynamic analysis of the cutting unit, although they are indispensable for physical assembly. Consequently, the algorithm maintains an active part whitelist to automatically retrieve and remove all redundant entities not involved in the core transmission chain before generating the final simulation script. This approach substantially enhances simulation efficiency without compromising the global dynamic characteristics.

### 4.8. Python-driven generation of C# automated modeling scripts and efficiency validation for multiple shearer cutting unit models

**Fig 13:**
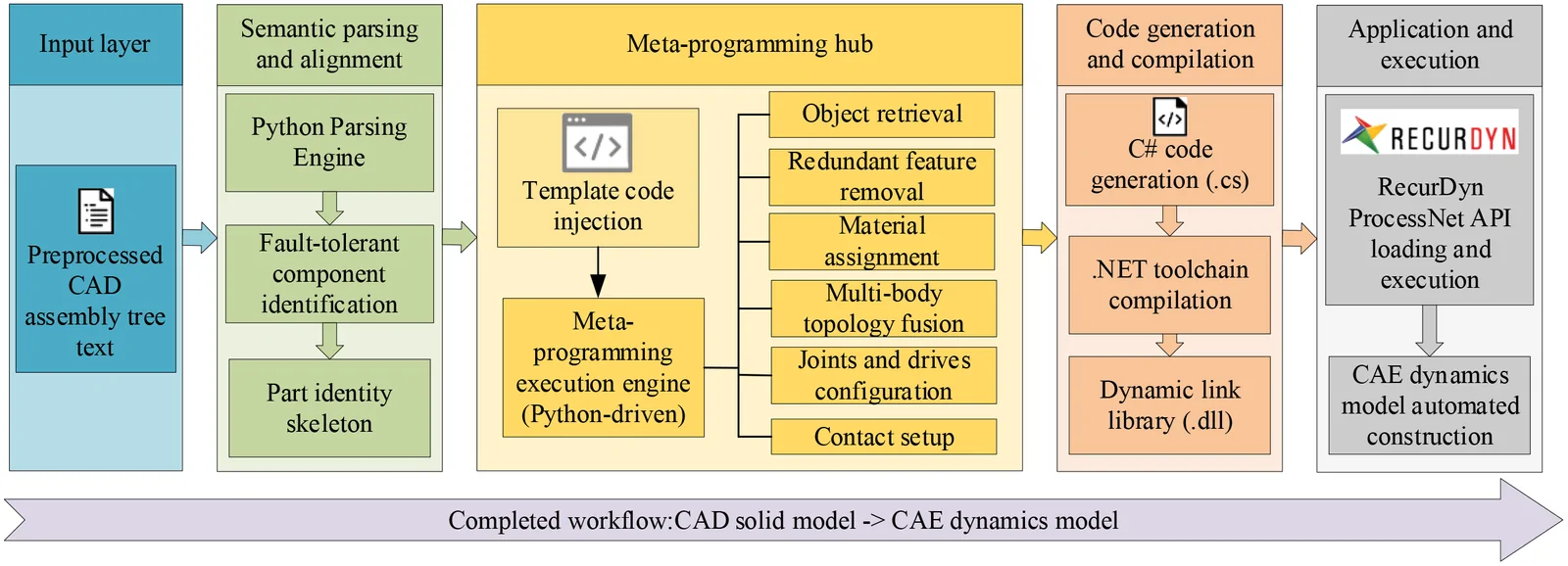
Methodology of the automated modeling execution engine based on meta-programming.

In this study, an automated modeling execution engine is developed based on the concept of meta-programming. The engine parses topological mapping tables via Python and utilizes placeholder injection to dynamically generate standard C# source code. Core modeling logic, including object retrieval, redundancy elimination, material assignment, topological fusion, constraints and drives, and contact settings, is automatically encapsulated within the scripts. The generated.cs scripts are compiled into.dll using the.NET toolchain, which are then directly loaded and executed by the RecurDyn software interface. The software environment comprised Python 3.12, Microsoft Visual Studio Professional 2022, and.NET Framework 4.8. This process establishes a cross-domain link from CAD assembly semantic analysis to the automated construction of CAE dynamic models. The schematic of the entire automated modeling process is shown in Fig 13.

**Fig 14:**
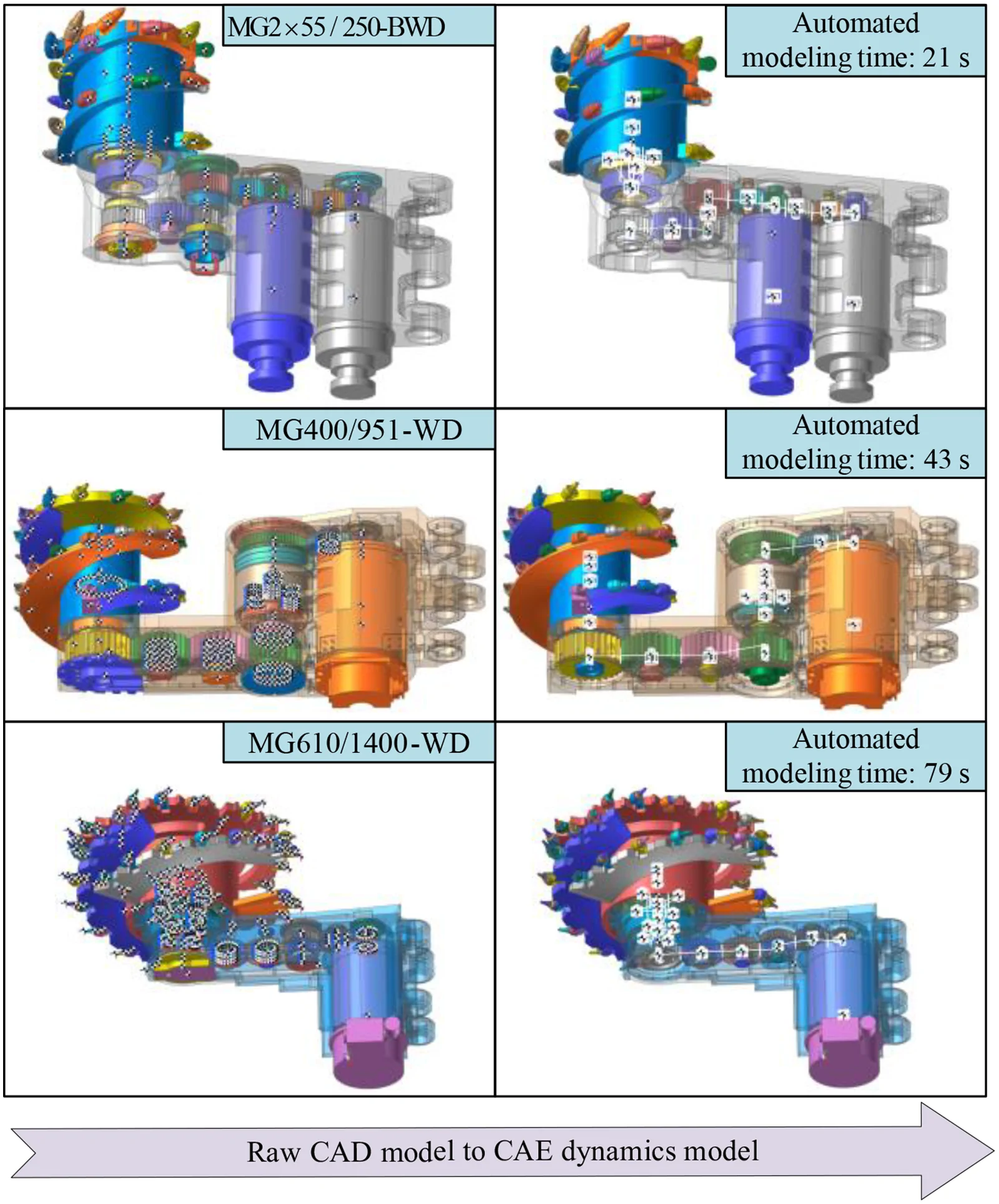
Automated modeling outcomes for three types of shearer cutting units with varying structural complexities.

All automated modeling efficiency tests were conducted on a desktop computer equipped with an AMD Ryzen 7 5800X 8-Core CPU and 64 GB of RAM, running Windows 10. To validate the universality, robustness, and efficiency of the proposed automated modeling methodology, modeling tests were conducted on three types of shearer cutting units with varying structural complexities. These units are designed for thin, medium-thick, and thick coal seams, respectively, and correspond to the models MG2 × 55/250-BWD, MG400/951-WD, and MG610/1400-WD. The resulting automated modeling outcomes for these systems are illustrated in Fig 14.

As illustrated in Fig 14, the imported raw CAD model contains a vast number of unprocessed discrete entities and redundant features, which are shown on the left side of the figure. Following the execution of the automated script, the system successfully achieves the autonomous construction of the MBD model, as presented on the right side. Detailed modeling data are provided in Table 4.

**Table 4:** Detailed automated modeling data for three types of shearer cutting units.

Shearer model	Original CAD part count	Effective part count after automation	Number of joints	Number of contacts	Automation modeling time(s)
MG2 × 55/250-BWD	166	32	33	15	21
MG400/951-WD	637	29	33	11	43
MG610/1400-WD	1041	39	39	21	79

Table 4 summarizes the assembly scale and modeling duration for the three cutting units. The reported modeling times are the average values obtained from five repeated runs for each model under the same hardware and software conditions. Taking the MG610/1400-WD, the largest model, as an example, its raw CAD model consists of 1041 initial parts. After topological fusion and redundancy elimination, the system simplifies the assembly into 39 effective components. It then automatically identifies and generates 39 constraints and 21 contact pairs, with the entire modeling workflow completed in just 79 s. Additionally, for the smaller MG400/951-WD and MG2 × 55/250-BWD models, the automated modeling times are 43 s and 21 s, respectively. These results demonstrate that the algorithm maintains high-efficiency computational performance across various levels of model complexity, highlighting its excellent robustness.

Traditional interactive modeling is heavily reliant on engineering experience, particularly when dealing with nested structures like multi-stage planetary gear trains where manual configuration is prone to constraint omissions or positioning deviations, resulting in an average processing time of approximately 180 min. In stark contrast, the proposed method reduces the modeling duration to less than 1% of the manual mode. While avoiding human errors and drastically improving efficiency, this methodology provides a highly efficient and reliable new paradigm for the fully automated evolution of complex equipment from CAD geometries to CAE dynamic models.

## 5. Simulation analysis of the shearer

### 5.1. Kinematic simulation analysis of the rigid body model

**Fig 15:**
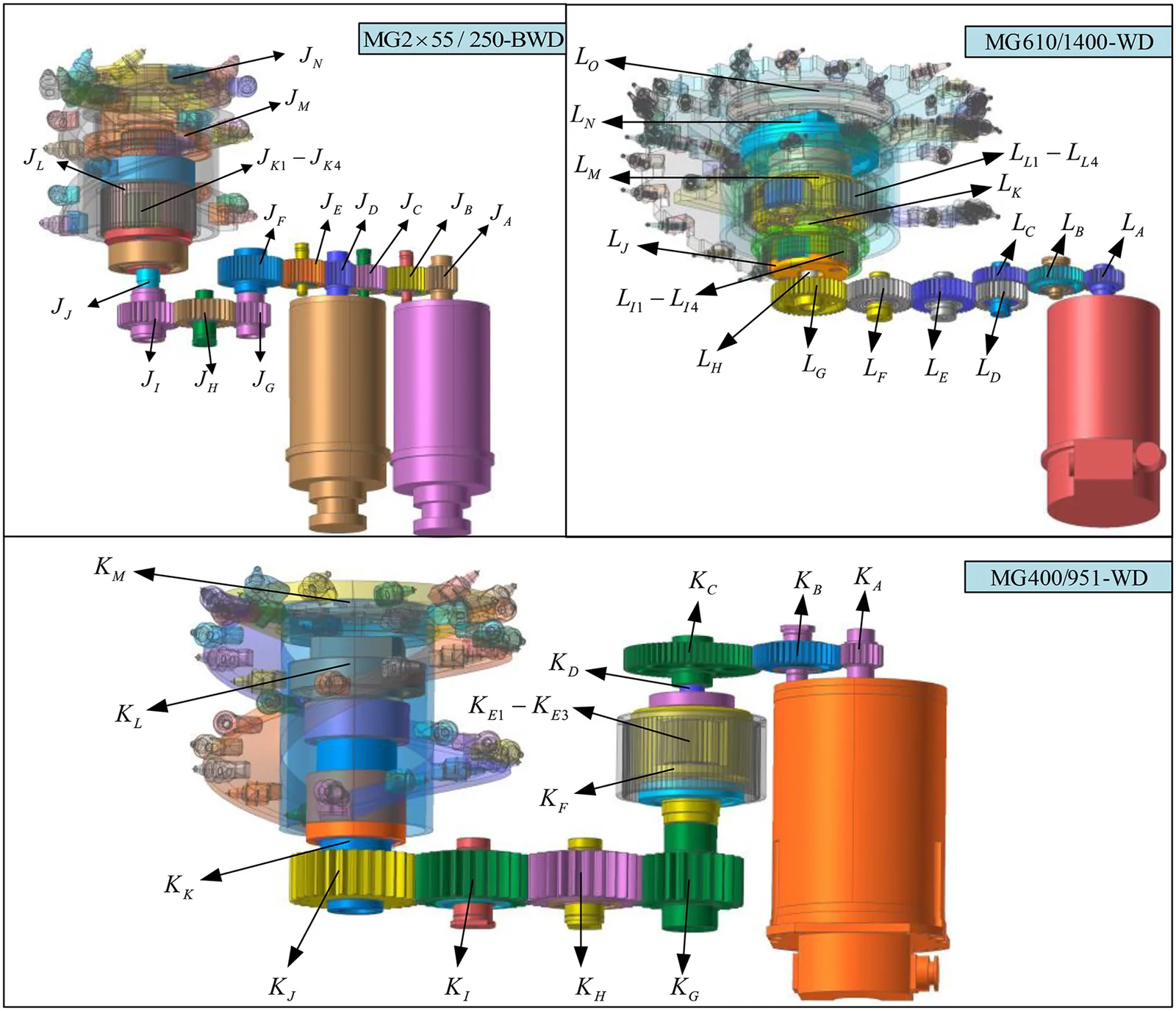
Transmission system diagram of cutting unit.

To verify the accuracy of the motion relationships among the components of the shearer’s rigid body model, a kinematic analysis was conducted. During the virtual prototype simulation, only gravitational forces were applied, with no dynamic loads imposed on the drum. For all three cutting unit models, the motor speed input was defined as Step(time, 0, 0, 0.1, 8880d), where d denotes degrees in RecurDyn and 8880°/s corresponds to 1480 r/min. The transmission systems of three different shearer cutting unit models are illustrated in Fig 15.

The planetary reducers within the transmission chains of the shearer cutting units in Fig 15 are all of the 2K-H type. The transmission ratio is calculated using the following formula [22]:


$$\begin{array}{c} i_{h}=1+\frac{z_{a}}{z_{b}} \end{array}$$ (4)

Where, *i*_*h*_ is the speed ratio between the planet carrier and the sun gear, *z*_*a*_ is the number of teeth on the internal ring gear, *z*_*b*_ is the number of teeth on the sun gear.

Except for the planetary reducer, all other gears are cylindrical spur gears. The transmission ratio is calculated using the following formula:


$$\begin{array}{c} i_{12}=z_{\text{2}}/z_{\text{1}} \end{array}$$ (5)

Where, *i*_12_ is the transmission ratio between two adjacent gears, *z*_1_ and *z*_2_ are the numbers of teeth of the two adjacent gears.

**Fig 16:**
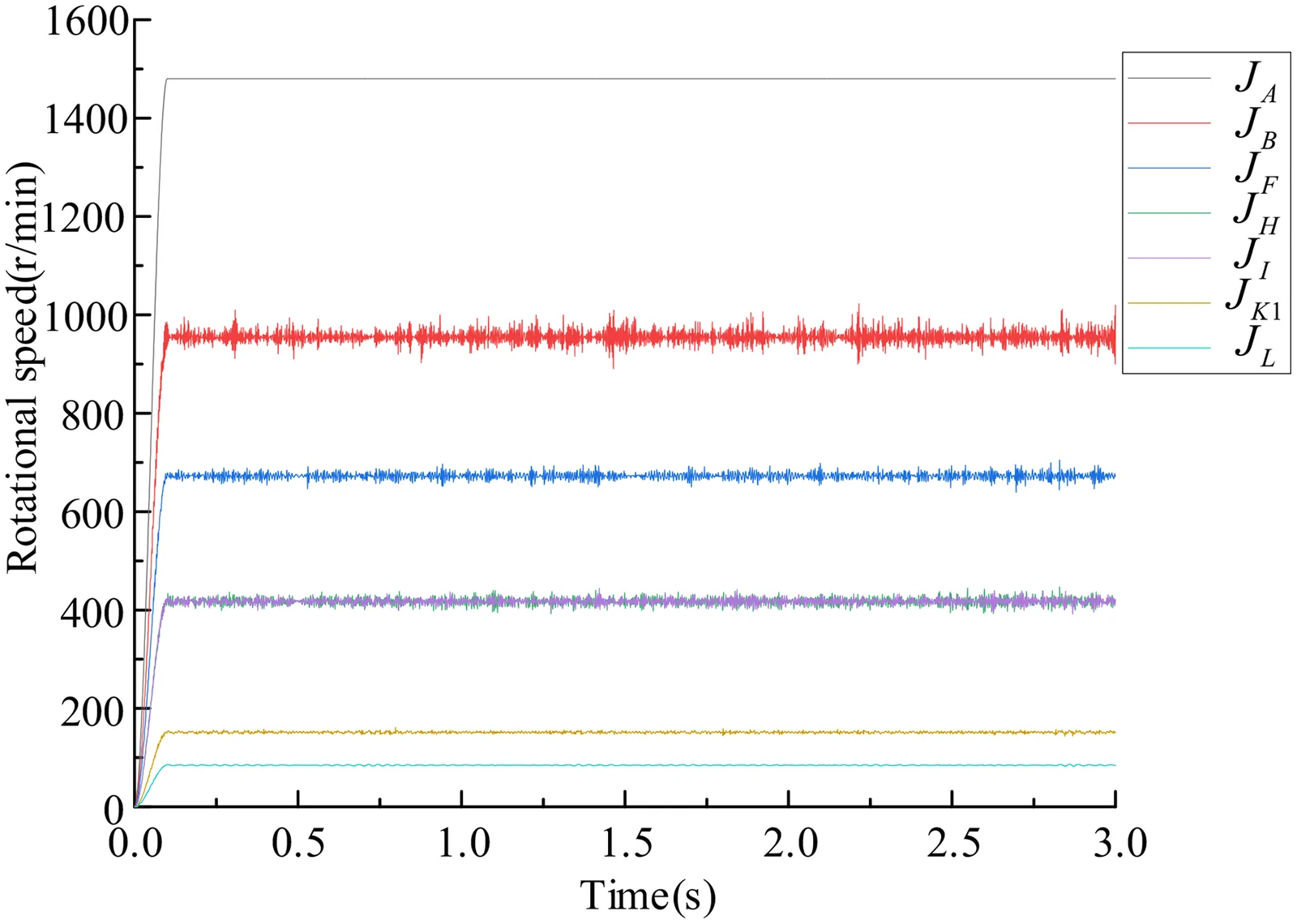
Speed curves of transmission components in the cutting unit of MG2 × 55/250-BWD shearer.

**Fig 17:**
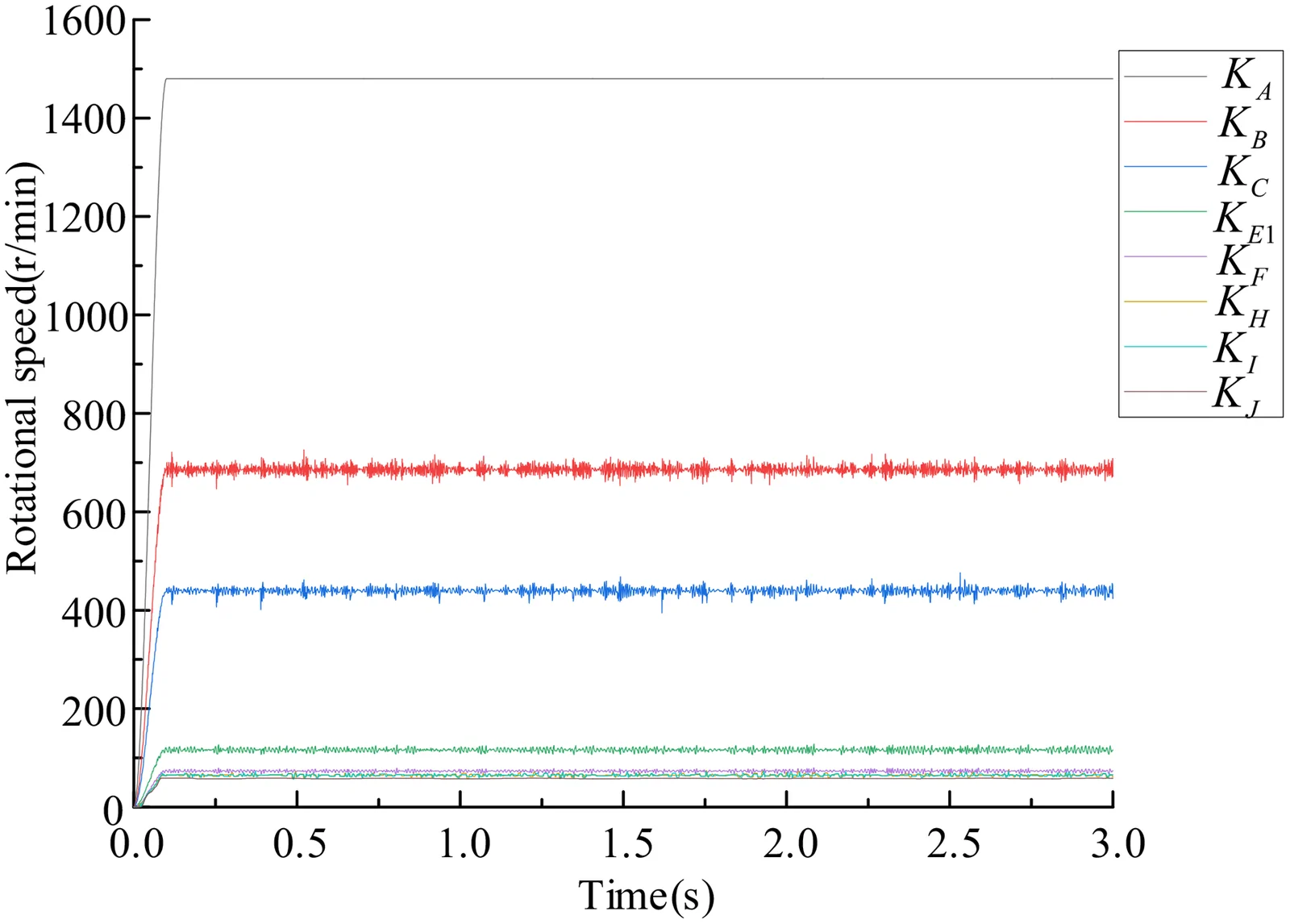
Speed curves of transmission components in the cutting unit of MG400/951-WD shearer.

**Fig 18:**
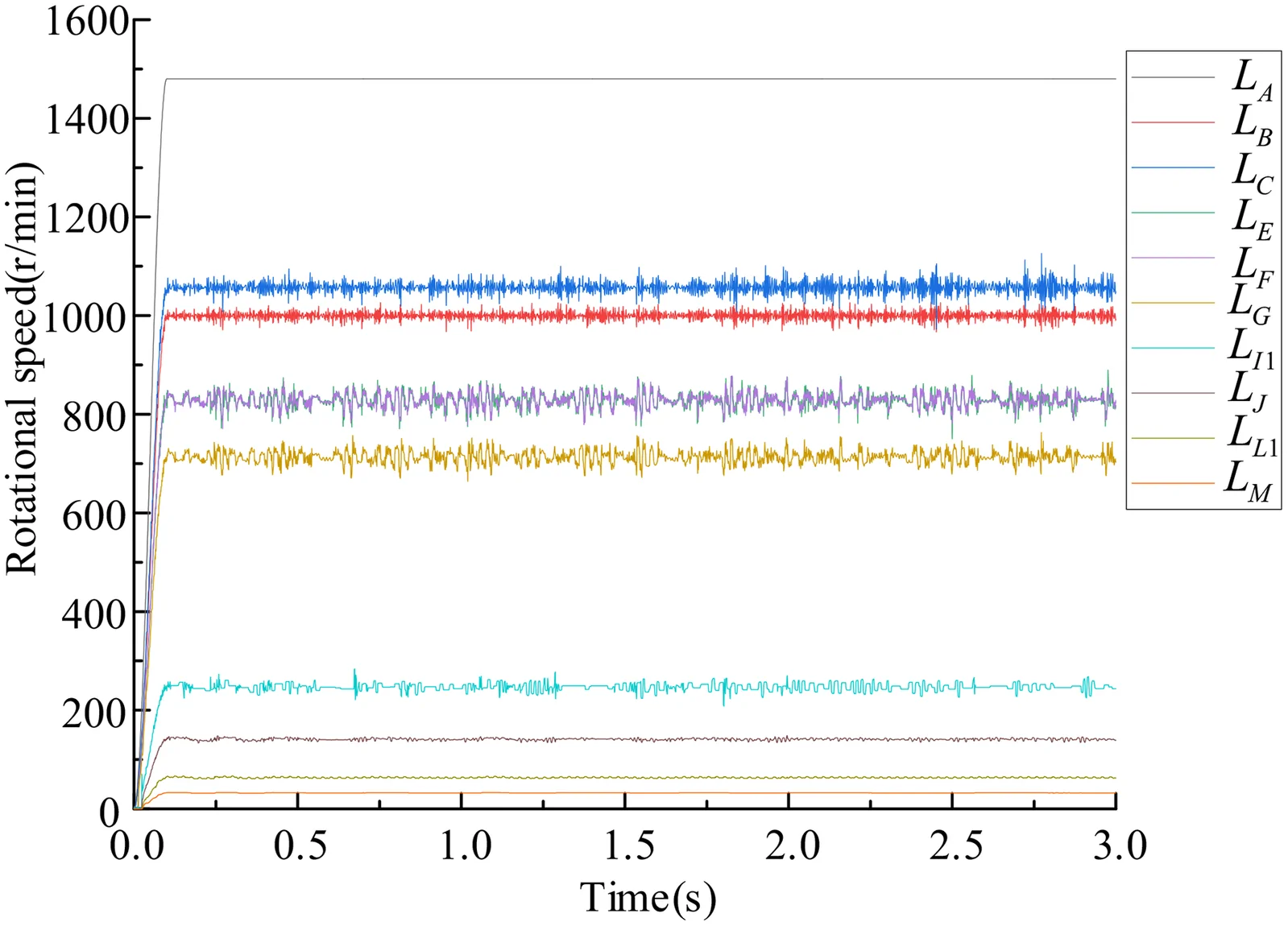
Speed curves of transmission components in the cutting unit of MG610/1400-WD shearer.

The cutting unit of the MG2 × 55/250-BWD shearer is driven by dual motors, with gears *J*_*B*_, *J*_*C*_, *J*_*E*_ having identical numbers of teeth, gears *J*_*F*_ and *J*_*G*_ being coaxial. Gear *J*_*I*_ and the sun gear *J*_*J*_ are fixed together via a rigid joint, and the planetary carrier *J*_*L*_ is connected to the drum *J*_*N*_ through a square shaft. This results in equal rotational speeds for gears *J*_*A*_ and *J*_*D*_; gears *J*_*B*_, *J*_*C*_, and *J*_*E*_ rotating at the same speed; gears *J*_*F*_ and *J*_*G*_ sharing the same rotational speed; and the planetary carrier *J*_*L*_ rotating at the same speed as the drum *J*_*N*_. Therefore, the rotational speeds of components *J*_*A*_, *J*_*B*_, *J*_*F*_, *J*_*H*_, *J*_*I*_, *J*_*K*1_,and *J*_*L*_ in the cutting unit of the MG2 × 55/250-BWD shearer are specifically recorded, as shown in Fig 16. Similarly, for the cutting unit of the MG400/951-WD shearer, only the rotational speeds of components *K*_*A*_, *K*_*B*_, *K*_*C*_, *K*_*E*1_, *K*_*F*_, *K*_*H*_, *K*_*I*_, and *K*_*J*_ are recorded, as shown in Fig 17. Likewise, for the MG610/1400-WD shearer, only the rotational speeds of components *L*_*A*_, *L*_*B*_, *L*_*C*_, *L*_*E*_, *L*_*F*_, *L*_*G*_, *L*_*I*1_, *L*_*J*_, *L*_*L*1_,and *L*_*M*_ are recorded,as shown in Fig 18.

The actual rotational speeds *n*_*0*_ of the transmission components during the stable simulation phase were obtained using RecurDyn’s post-processing module, and the theoretical speeds *n*, were calculated using Eqs. (4) and (5), as presented in Table 5.

**Table 5:** Rotational speeds of all drive components in the shearer cutting unit.

Shearer model	Designation	Theoretical rotational speed *n* (r/min)	Actual rotational speed *n*_*0*_ (r/min)	Relative error
MG2 × 55/250-BWD	J_A_	1480.000	1480.000	0.000%
	J_B_	954.839	954.879	0.004%
	J_F_	672.727	672.697	0.004%
	J_H_	417.555	417.496	0.014%
	J_I_	417.555	417.580	0.006%
	J_K1_	151.405	151.406	0.001%
	J_L_	84.505	84.507	0.002%
MG400/951-WD	K_A_	1480.000	1480.000	0.000%
	K_B_	685.854	685.938	0.012%
	K_C_	439.375	439.383	0.002%
	K_E1_	116.156	116.158	0.002%
	K_F_	73.229	73.229	0.000%
	K_H_	64.780	64.787	0.011%
	K_I_	64.780	64.791	0.017%
	K_J_	58.078	58.089	0.019%
MG610/1400-WD	L_A_	1480.000	1480.000	0.000%
	L_B_	1000.000	1000.010	0.001%
	L_C_	1057.143	1057.273	0.012%
	L_E_	828.571	828.518	0.006%
	L_F_	828.571	828.475	0.012%
	L_G_	712.957	712.897	0.008%
	L_I1_	246.936	246.990	0.022%
	L_J_	141.106	141.131	0.018%
	L_L1_	63.380	63.394	0.022%
	L_M_	32.337	32.345	0.025%

Figs 16–18 show that, during the 0.1–3 s interval, the actual rotational speeds of the transmission components fluctuate steadily around their theoretical values. As shown in Table 5, the maximum relative error is 0.025%, and the relative errors of other transmission components are all below 0.1%. Therefore, the gear meshing transmission among the shearer’s components is verified to be accurate, confirming the correctness of the constraints and drives applied between parts in the virtual prototype model developed through RecurDyn secondary development. Building on this, the MG2 × 55/250-BWD shearer is selected as a representative case to further conduct complex dynamic simulations and comparative experimental studies.

### 5.2. Dynamic simulation analysis of the Rigid–Flexible coupling model based on DEM–MFBD bidirectional coupling technology

**Fig 19:**
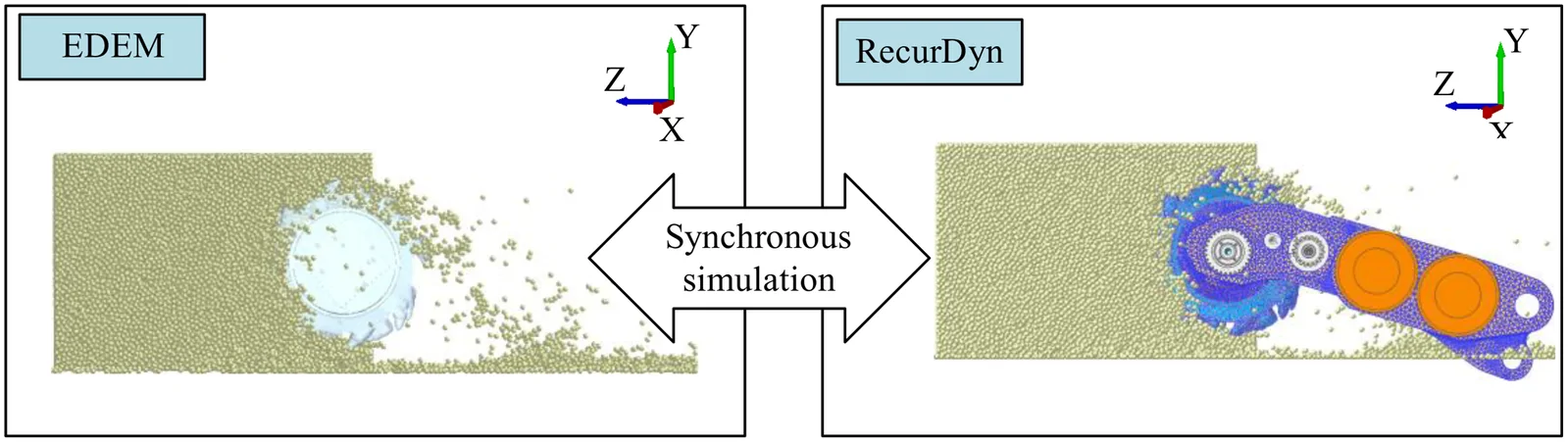
EDEM−RecurDyn bidirectional coupling coal cutting state at a certain time.

After verifying the kinematic characteristics of the gear transmission, key components, including the helical drum, cutting unit housing, and planetary carrier, are modeled as flexible bodies to more accurately simulate the dynamic behavior during the cutting process and validate the reliability of the model. On this basis, the Discrete Element Method–Multi Flexible Body Dynamics (DEM-MFBD) bidirectional coupling technique is employed to facilitate transient data interaction between EDEM and RecurDyn via the External SPI interface and wall format files [23]. During each time step, RecurDyn transmits positional data to EDEM to update the geometry; EDEM then calculates the particle motion and resultant interaction forces, which are fed back to RecurDyn to update the model’s motion state [24]. Since the drum functions to load and discharge coal and remains coupled with the coal wall throughout operation [25], it serves as the primary medium for this data exchange, as illustrated in Fig 19.

In RecurDyn, the traction speed of the shearer was set to 3 m/min, and the rotational speed of the drum was set to 84.5 r/min. The total simulation time was 3 s. To ensure that the collected vibration data accurately reflected the cutting state, the sampling frequency was set to 2000 Hz according to the sampling theorem [26], and the simulation step size was 0.0005 s.

**Fig 20:**
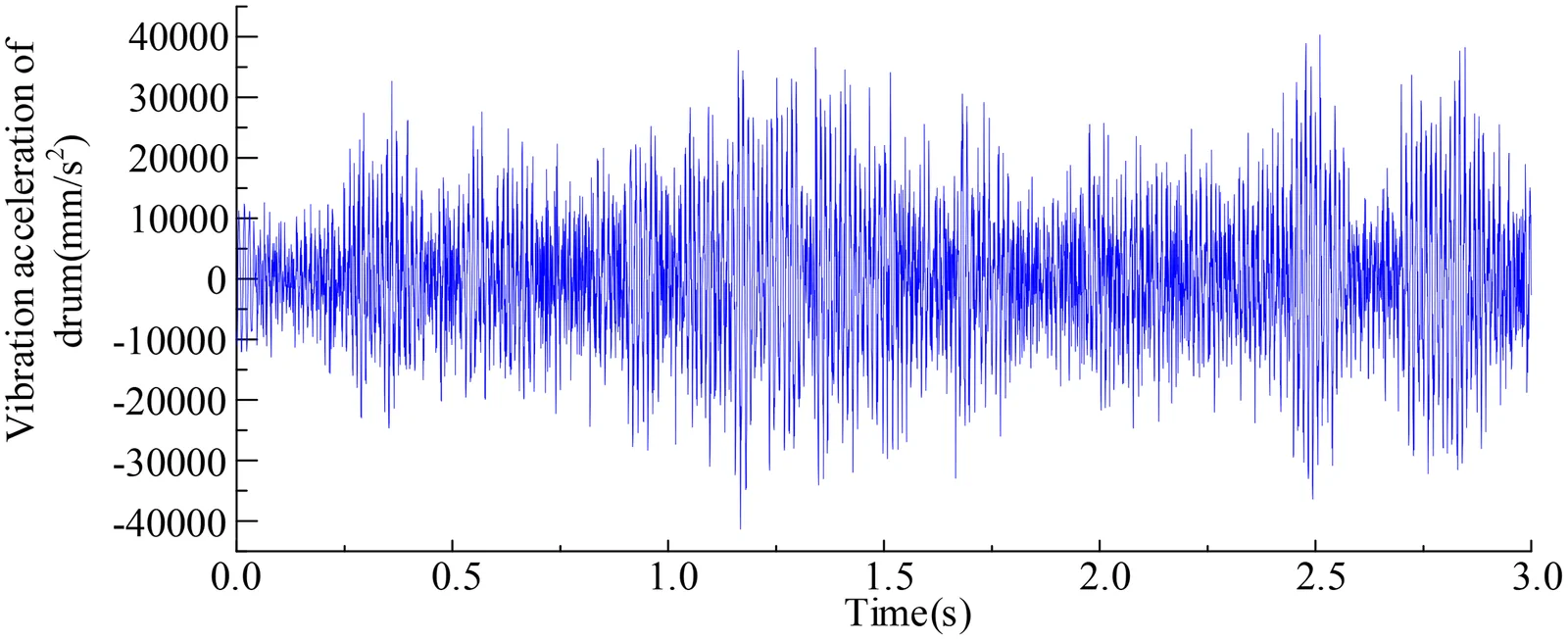
Drum vibration characteristics based on simulation.

**Fig 21:**
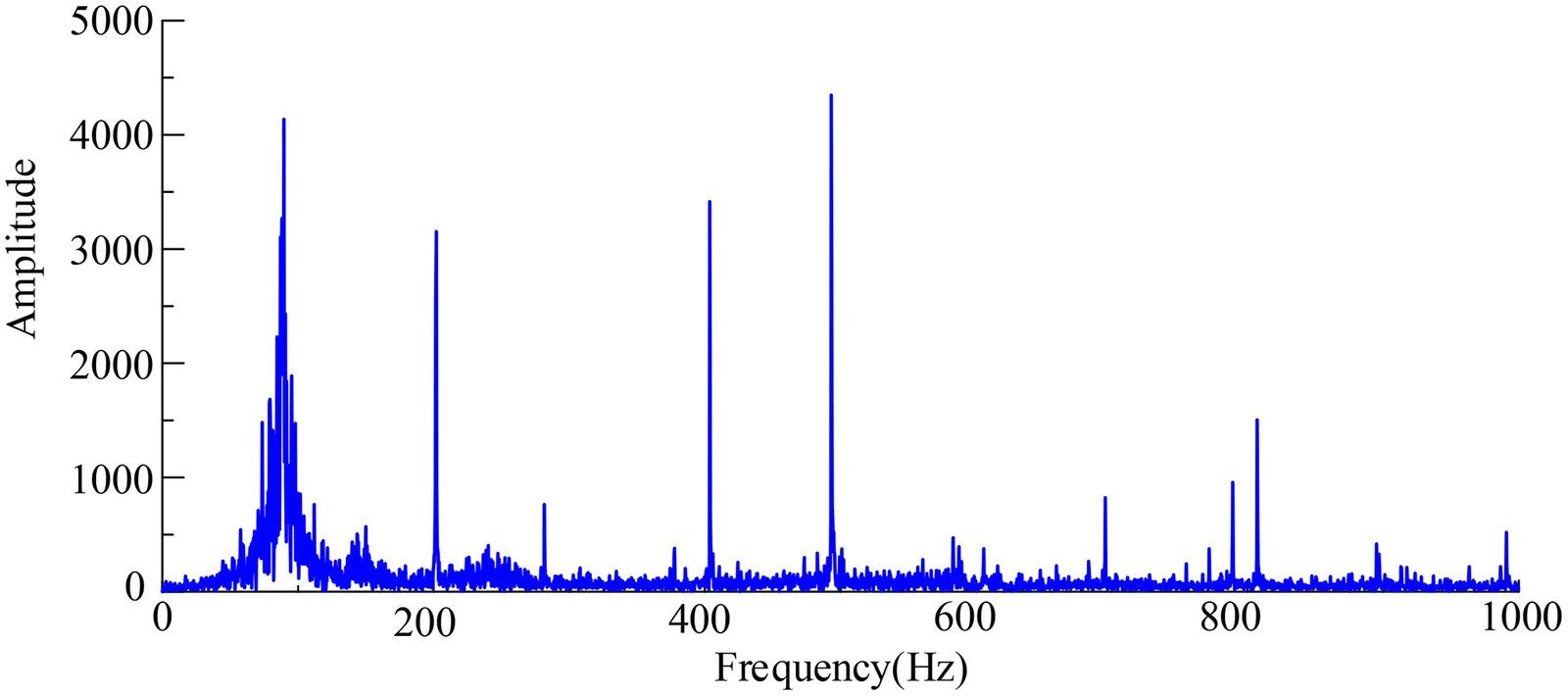
Signal amplitude spectrum based on simulation.

The time-domain and frequency-domain signals of the vibration acceleration in the direction of the drum cutting resistance are illustrated in Fig 20 and Fig 21, respectively.

Due to the nonlinear, time-varying, and strongly coupled characteristics of the loads acting on the shearer’s drum, significant vibrations are easily induced during the cutting process. Statistical analysis reveals that the Root Mean Square (RMS) value of the time-domain signal in Fig 20 is 11022.4 mm/s^2^. Furthermore, as indicated by Fig 21, the dominant frequencies during coal cutting are primarily distributed around 88, 202, 404, and 493 Hz.

## 6. Experimental testing of shearer vibration signals based on similarity theory

**Fig 22:**
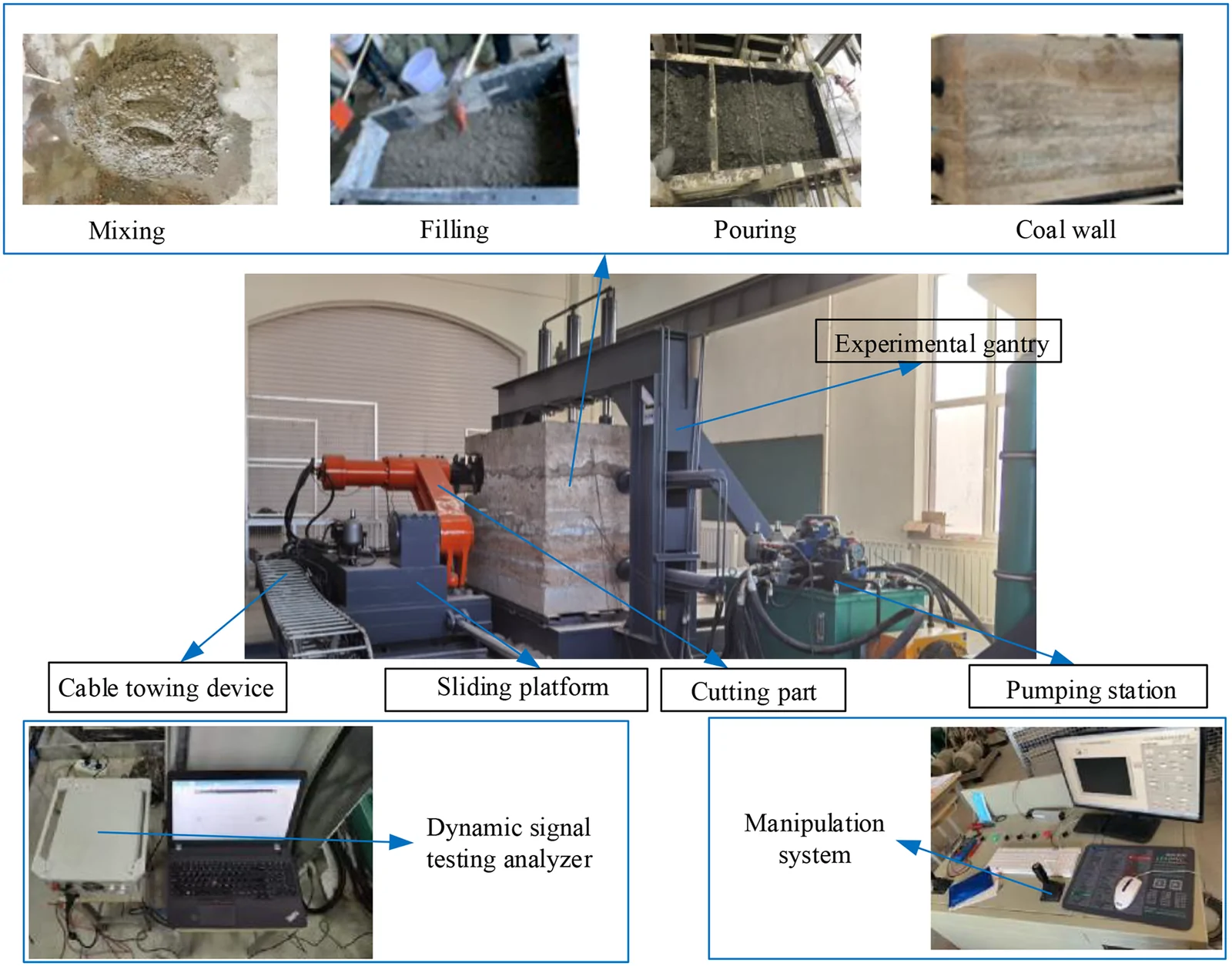
Shearer cutting test platform.

To verify the effectiveness of the research methodology and results, a shearer helical drum cutting experiment was conducted in the State Key Laboratory of Large Mining Equipment. Owing to the large structural dimensions of the shearer and the complex working conditions of the coal wall, constructing a full-scale physical model is highly challenging. Consequently, an equivalent experimental platform was developed based on similarity theory [27]. The platform primarily comprises a cutting unit, an artificial simulated coal wall, and a sliding platform. To ensure parameter equivalence between the experimental system and the virtual simulation environment, a vibration acceleration sensor was installed on the drum to monitor cutting vibration signals in real-time via a data acquisition system. The structure of the experimental platform is illustrated in Fig 22.

**Fig 23:**
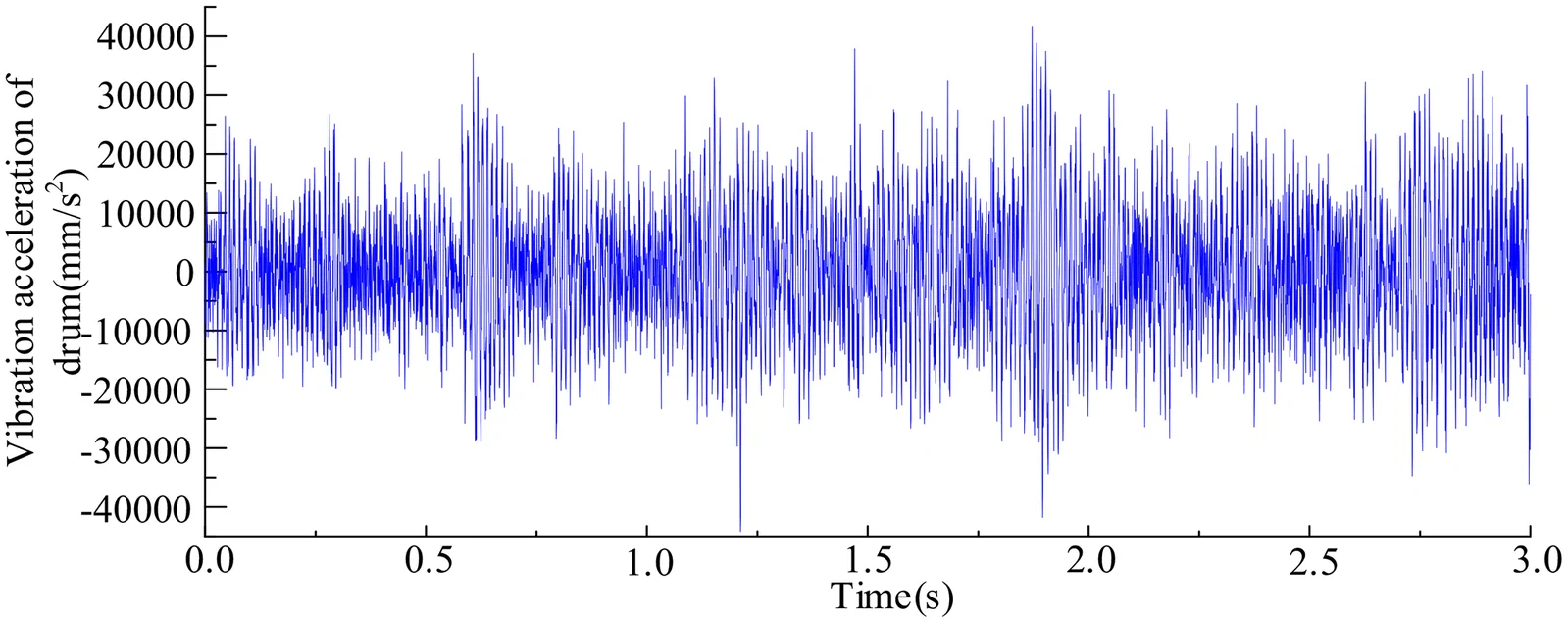
Drum vibration characteristics based on test.

**Fig 24:**
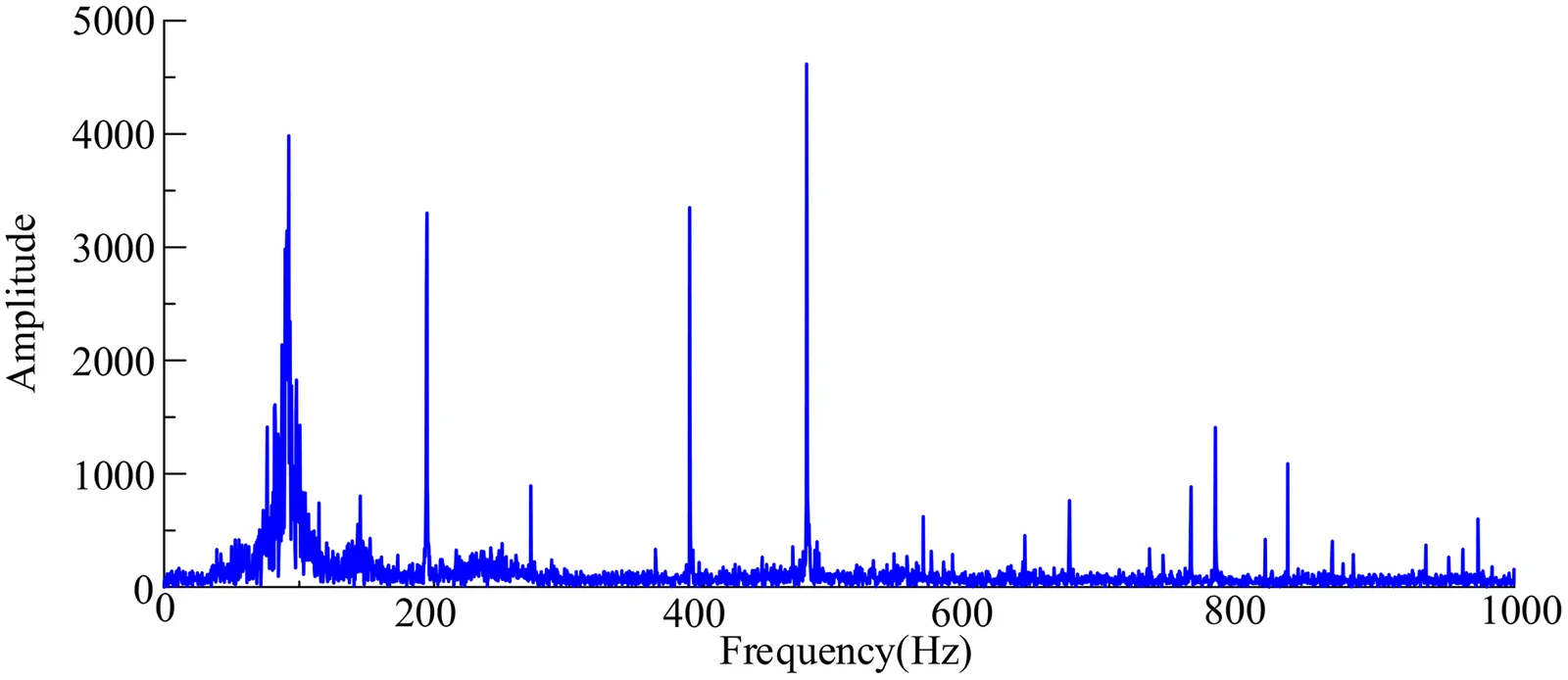
Signal amplitude spectrum based on test.

The signals from the vibration acceleration sensor were acquired using a signal testing and analysis instrument. After conversion according to the similarity ratios, the time-domain and frequency-domain signals of the vibration acceleration in the direction of the drum cutting resistance are illustrated in Fig 23 and Fig 24, respectively.

As shown in Fig 23 and Fig 24, the vibration responses of the drum during coal-wall cutting exhibit only minor deviations from the results of the DEM-MFBD numerical simulation. Statistical analysis reveals that the RMS value of the time-domain signal in Fig 23 is 10517.9 mm/s^2^. Furthermore, as indicated by Fig 24, the dominant frequencies during coal cutting are primarily distributed around 86, 194, 389, and 475 Hz. The relative error of the RMS values between the simulated and experimental time-domain signals is only 4.8%, while the maximum error for the frequency-domain signals is just 4.1%. In summary, the feasibility of the simulation and the reliability of the results are thoroughly validated from both time-domain and frequency-domain perspectives.

## 7. Conclusion

To address the inefficiency and high error rates of manual multibody system modeling, a rapid automated modeling methodology was developed based on meta-programming with auxiliary Transformer-assisted semantic matching for fault-tolerant component identification using the RecurDyn PNet platform. The validity of the generated dynamic models was comprehensively verified through motion analysis of a gear transmission system and vibration response analysis of a dynamic drum. The primary conclusions are as follows:

(1) By integrating a fault-tolerant component identification strategy with Python-driven meta-programming for automated C# code generation, an automated modeling methodology was established for the rapid construction of MBD models. Verification on shearer cutting units for thin, medium-thick, and thick coal seams demonstrates that the methodology achieves automated mapping from CAD assembly data to dynamic entities in less than 1% of the time required for traditional manual modeling. This proves the method’s feasibility and efficiency in significantly reducing modeling cycles and facilitating subsequent structural optimization.
(2) Kinematic simulations conducted on three shearer cutting units with different structures (thin, medium-thick, and thick coal seams) show that the maximum relative error between simulated and theoretical rotational speeds is 0.025%, with all errors remaining below 0.1%. These results verify the correctness and generality of the automatically generated models, providing a reliable tool for the rapid design and dynamic analysis of shearer cutting units across various coal seam conditions.
(3) Bidirectional DEM-MFBD coupling was utilized to simulate coal-rock cutting processes, where the simulated drum vibration signals align closely with experimental results. The RMS error of vibration acceleration is 4.8%, and the maximum relative error of primary characteristic frequencies is 4.1%. These results further validate the accuracy and robustness of the automated models under complex dynamic load conditions.
